# Natural variations of *FT* family genes in soybean varieties covering a wide range of maturity groups

**DOI:** 10.1186/s12864-019-5577-5

**Published:** 2019-03-20

**Authors:** Bingjun Jiang, Shouwei Zhang, Wenwen Song, Mohammad Abdul Awal Khan, Shi Sun, Chengsheng Zhang, Tingting Wu, Cunxiang Wu, Tianfu Han

**Affiliations:** MARA Key Laboratory of Soybean Biology (Beijing), Institute of Crop Science, the Chinese Academy of Agricultural Sciences, 12 Zhongguancun South Street, Beijing, 100081 China

**Keywords:** Soybean, *FT* family genes, Flowering time, Maturity group, Haplotype

## Abstract

**Background:**

Flowering time and maturity are among the most important adaptive traits in soybean (*Glycine max* (L.) Merill). *Flowering Locus T* (*FT*) family genes function as key flowering integrators, with flowering-promoting members *GmFT2a/GmFT5a* and flowering-inhibiting members *GmFT4*/*GmFT1a* antagonistically regulating vegetative and reproductive growth. However, to date, the relations between natural variations of *FT* family genes and the diversity of flowering time and maturity in soybean are not clear. Therefore, we conducted this study to discover natural variations in *FT* family genes in association with flowering time and maturity.

**Results:**

Ten *FT* family genes, *GmFT1a*, *GmFT1b*, *GmFT2a*, *GmFT2b*, *GmFT3a*, *GmFT3b*, *GmFT4*, *GmFT5a*, *GmFT5b* and *GmFT6*, were cloned and sequenced in the 127 varieties evenly covering all 14 known maturity groups (MG0000-MGX). They were diversified at the genome sequence polymorphism level. *GmFT3b* and *GmFT5b* might have experienced breeding selection in the process of soybean domestication and breeding. Haplotype analysis showed that a total of 17 haplotypes had correlative relationships with flowering time and maturity among the 10 *FT* genes, namely, 1a-H3, 1b-H1, 1b-H6, 1b-H7, 2a-H1, 2a-H3, 2a-H4, 2a-H9, 2b-H3, 2b-H4, 2b-H6, 2b-H7, 3b-H4, 5a-H1, 5a-H2, 5a-H4 and 5b-H1. Based on the association analysis, 38 polymorphic sites had a significant association with flowering time at the level of *p* < 0.01.

**Conclusions:**

Some natural variations exist within the 10 *FT* family genes, which might be involved in soybean adaptation to different environments and have an influence on diverse flowering time and maturity. This study will facilitate the understanding of the roles of *FTs* in flowering and maturity.

**Electronic supplementary material:**

The online version of this article (10.1186/s12864-019-5577-5) contains supplementary material, which is available to authorized users.

## Background

Soybeans are planted within a wide range of latitudes across the world resulting from the rich diversity of varieties in flowering time and maturity. In North America, soybeans are classified into 13 maturity groups: MG000 to MGX in the ascending order of maturity [1]. Gai divided 256 Chinese soybean landraces into 12 maturity groups without MGX [2]. MG0000, a new maturity group that matures the earliest to date was identified in some super-early varieties from high-latitude cold regions in China and the Far East of Russia [3]. Previous research indicates that maturity diversity in soybean is attributed to the variation and combination of genes responding to photoperiod and temperature [3–6].

Multiple genes control flowering time and maturity in soybean. A total of 10 maturity loci are characterized in soybean, including *E1*-*E10* and *J* [7–16]. A majority of these loci were reviewed by Xia et al. [17]. They play different roles in flowering and maturity maintenance under diverse photoperiod patterns. To date, six of these loci, *E1* [18], *E2* [19], *E3* [20], *E4* [21], *E9* [22, 23] and *J* [24, 25], have been cloned. Among the six loci, *E9* has been confirmed as *GmFT2a*, an orthologue of the Arabidopsis *Flowering Locus T* (*FT)* gene, which plays an important role in flowering [22, 23, 26].

*FT,* which encodes a putative florigen, is a key integrator gene in the regulation of flowering in Arabidopsis [27], and the function is highly conserved in different species [28, 29]. Ten *FT-like* genes have been identified in soybean among which *GmFT2a* and *GmFT5a* are confirmed as flowering promoters; whereas *GmFT1a* is divergent as a floral and maturity inhibitor [26, 30, 31]. *GmFT2a* and *GmFT5a* coordinately control flowering as flowering integrators and can be regulated by *E1*, *E2*, *E3* and *E4* [18, 19, 26, 32]. Ectopic expression experiments in Arabidopsis also demonstrated that other soybean *FT* family genes including *GmFT2b*, *GmFT3a*, *GmFT3b* and *GmFT5b* promoted flowering [33, 34], whereas *GmFT4* delayed flowering [35]. In transgenic soybean overexpressing *E1*, *GmFT1a* and *GmFT4* expression was up regulated, whereas the expression of *GmFT2a* and *GmFT5a* was suppressed [31, 35]. Currently, a new *GmFT2c* was recently found inserted in a putative transponson in the third intron, and *GmFT2d* was determined structurally rearranged with some remnant in the genome, indicating that *GmFT2* subclades (*GmFT2a*, *GmFT2b*, *GmFT2c* and Gm*FT2d*) have different evolutionary trajectories [36].

In plants, multiple regulation pathways regulate *FT* expression in response to diverse environmental signals, which enable flowering and other developmental responses to be seasonally timed [37]. Different natural variations of *FT* promoter sequences are confirmed responsible for diverse flowering time in Arabidopsis and also in rice [38, 39]. The promoter of *GmFT2a* in soybean has high polymorphism, and some SNPs in the promoter region are associated with flowering time and photoperiod sensitivity [34]. *GmFT2a* has a recessive allele for delayed flowering, which contains a *Ty1*/*copia*-like retrotransposon SORE-1 inserted in the first intron that attenuates *GmFT2a* expression by its allele-specific transcriptional repression [23]. Critical sequence polymorphism across the gene and its flanking regions may reflect the evolutionary process of species adapting to different environments. The *FT* homologs have functional divergence in soybean [22, 23, 26, 31, 32], implying that *FT* genes play roles that influence the flowering and maturation time through an interactive balance when receiving different environmental signals. Therefore, whether their natural variations are linked to flowering time and maturity requires investigation.

In this study, 127 soybean varieties covering all known 14 maturity groups (MG) from MG0000 to MGX were selected and grown for phenotypic and genotypic identification. The sequence polymorphisms of 10 soybean *FT* family genes in all varieties were analyzed. Further analysis of *FT* haplotypes and their natural variations associated with flowering time and maturity was conducted. The results suggested that some natural variations of the 10 soybean *FT* genes existed in soybeans of different MGs and that these variations were related to soybean flowering time and maturity. Our findings will elucidate the roles of soybean *FT* family genes in flowering and maturity.

## Methods

### Plant materials and investigation of flowering time and maturity

One hundred and twenty-seven soybean (*Glycine max*) varieties covering all known 14 maturity groups were collected for the experiment (Additional file 1: Table S1). These varieties were collected from the Institute of Crop Science, the Chinese Academy of Agricultural Sciences, which are public and available for non-commercial purpose. They were primarily from China and North America, with a few from Brazil, India, Costa Rica and the Far East of Russia. Fifteen soybean seeds were planted in 10-l pots on May 27, 2015, and grown outdoors under natural conditions in Beijing, China (39.95° N, 116.32° E). After Ve (emergence), seedlings with similar size were selected so that each pot contained five uniform plants. Each variety was planted in three replicates pots. We collected data on the developmental stages, including Ve, R1 (beginning bloom), R7 (physiological maturity) and R8 (full maturity), according to the description by Fehr and Carviness [40]. The mean of the three replications for each variety was used for statistical analysis.

### DNA isolation, PCR and sequencing

Genomic DNA was extracted from fresh trifoliate leaves using the standard cetyltrimethyl ammonium bromide (CTAB) method [41]. Seventeen PCR primer pairs were used to amplify 10 soybean *FT* family genes, and 17 fragments were produced. *GmFT1a*, *GmFT2a* and *GmFT6* were fragmentally amplified with three primer pairs, *GmFT3a* with two primer pairs, and *GmFT1b*, *GmFT2b*, *GmFT3b*, *GmFT4*, *GmFT5a* and *GmFT5b* were each amplified with one primer pair. The sequences of these primers are listed in Additional file 2: Table S2. Target regions were amplified with the high-fidelity polymerase of KOD-Plus-Neo and KOD-FX, produced by Toyobo Life Science, Beijing, China. Their reaction conditions are listed in Additional file 3: Table S3. The PCR products were directly sequenced using the Sanger method at Tsingke Biological Technology Company, Beijing, China.

### Sequence analysis and haplotype definition of the 10 soybean *FT* family genes

We used Seqman 1.0 for sequence assembly [42]. The sequences were aligned and adjusted manually using ClusterX 2.0 [43] and Bioedit [44]. The sequences after alignment were input into DnaSP v5 to calculate nucleotide diversity and Tajima’s D statistics [45]. The sequence variation ratio was calculated as follows: Sequence variation ratio = (No. of polymorphic sites/gene length, referring to the reference genome) × 100%. The phylogenetic relationships among different types of soybean FT proteins were analyzed using the neighbor-joining (NJ) method with the program MEGA 7 [46]. During the analysis of polymorphic sites, the 10 soybean *FT* family genes in soybean reference genome in the Phytozome database (https://phytozome.jgi.doe.gov/pz/portal.html) were used as references. We chose polymorphic sites with minor allele frequency above 4 varieties and imported them into Tassel 5 to analyze the linkage disequilibrium (LD) [47]. We conducted haplotype analysis based on sequence alignments and the feature of LD and different sites. After haplotype definition, the primary haplotypes of the 10 *FT* family genes were analyzed for association with flowering time and maturity. For those polymorphic sites that only appeared in these haplotypes with relation to flowering time and maturity, we defined them as candidate polymorphic sites (Additional file 4: Table S4). Then, we selected the candidate polymorphic sites of each soybean *FT* family gene and used Tassel 5 to identify polymorphic site-trait associations by generating a general linear model (GLM: trait = marker effect + residual) [48], and the cutoff of *p* < 0.01 is selected as in our previous study on *GmFT2a* promoter variations [34]. The polymorphic site information of the 10 soybean *FT* family genes is listed in Additional file 5: Table S5.

## Results

### Soybean varieties from different maturity groups varied greatly in days to R1, R7 and R8 from emergence

**Fig. 1:**
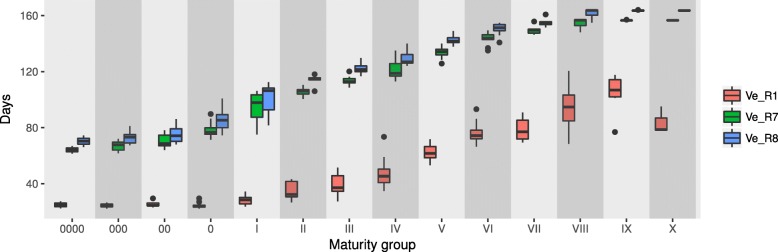
Ve-R1, Ve-R7 and Ve-R8 of 127 soybean varieties covering 14 maturity groups. Note: Ve-R1, Ve-R7 and Ve-R8 represent the days from emergence to the beginning of bloom, the physiological maturity and the full maturity, respectively

Except for some late-maturing varieties that did not reach R7 before the end of the experiment, the flowering time (Ve-R1) varied from 22.2 to 120.5 days, with a span of 98.3 days, and the maturity (Ve-R7) varied from 61.4 to 155.7 days, with a span of 94.3 days (Additional file 6: Table S6). Figure 1 shows that the Ve-R1 of the varieties from MG0000 to MG0 was similar under the natural photothermal conditions in Beijing, whereas the Ve-R1 gradually increased from MGI to MGX. Both Ve-R7 and Ve-R8 increased from MG0000 to MGX (Fig. 1). Among the tested varieties, MG000 variety Hujiao07–2479 flowered the earliest with the Ve-R1 of 22.2 days, and MG0000 variety Star4/75 matured the earliest with the Ve-R7 of 61.4 days (Additional file 6: Table S6). Zigongdongdou of MGVIII flowered the latest with the Ve-R1 of 120.5 days. The varieties exhibited rich diversity in flowering time and maturity.

### Sequence polymorphisms of the 10 soybean *FT* family genes

**Fig. 2:**
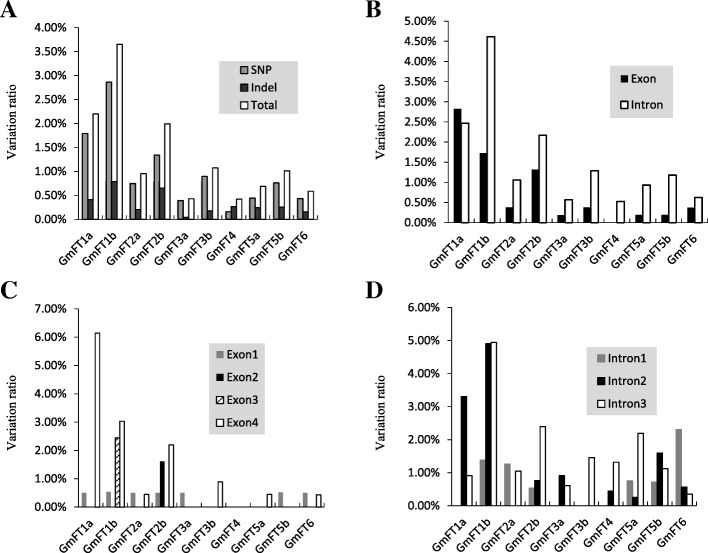
Sequence variation ratio analysis of the 10 *FT* family genes in soybean. **a**: Sequence variation ratio of SNPs and Indels. **b**: Sequence variation ratio of exons and introns. **c**: Sequence variation ratio of different exons. **d**: Sequence variation ratio of different introns

The results of sequence analysis showed that the 10 soybean *FT* family genes diversified in sequence polymorphism, with the polymorphism sites ranging from 8 (*GmFT4*) to 129 (*GmFT1a*) (Table 1). The π and θ_w_ of nucleotide diversity parameters extended from 0.00039 (*GmFT3a*) to 0.01102 (*GmFT1b*) and from 0.00080 (*GmFT3a*) to 0.00787 (*GmFT1b*), respectively (Table 1). For all the genes except *GmFT1a*, the variation ratios of introns were higher than those of exons. The variation ratio of introns in *GmFT1a* was slightly lower than that of exons (Fig. 2). Notably, variation ratios of exon4 of the 10 *FT* family genes were commonly higher than those of other exons, suggesting that the exon4 or its resultant protein domain was less conserved than other exons (Fig. 2).

**Table 1:** Summary of polymorphic sites of the 10 *FT* family genes in soybean

Gene	Sample size	No. of aligned bp	Type of mutation	No. of polymorphic sites	Sequence variation ratio	Nucleotide diversity		Tajima’s D
						π/bp	θw/bp	
*GmFT1a*	119	5957	SNP	105	1.79%	0.00434	0.0035	0.92498
			Indel	24	0.41%	0.00087	0.00096	−0.21635
*GmFT1b*	99	2745	SNP	73	2.86%	0.00845	0.00569	1.67398
			Indel	20	0.78%	0.00257	0.00218	0.54345
*GmFT2a*	118	5365	SNP	40	0.75%	0.0014	0.00133	0.15918
			Indel	11	0.21%	0.00072	0.00056	0.72832
*GmFT2b*	118	3058	SNP	41	1.34%	0.00385	0.00253	1.59284
			Indel	20	0.65%	0.00203	0.00129	1.67091
*GmFT3a*	122	2564	SNP	10	0.39%	0.00034	0.00073	−1.31769
			Indel	1	0.04%	0.00005	0.00007	−0.29027
*GmFT3b*	109	2241	SNP	20	0.89%	0.003	0.00169	2.20415*
			Indel	4	0.18%	0.00099	0.00042	2.84140**
*GmFT4*	127	1907	SNP	3	0.16%	0.00009	0.00029	−1.17378
			Indel	5	0.26%	0.00097	0.00068	0.99565
*GmFT5a*	127	2039	SNP	9	0.44%	0.0004	0.00084	−1.27444
			Indel	5	0.25%	0.0002	0.00047	−1.1339
*GmFT5b*	108	2942	SNP	21	0.76%	0.00349	0.00144	4.08832***
			Indel	7	0.25%	0.00112	0.00048	3.44592***
*GmFT6*	125	6545	SNP	28	0.43%	0.00033	0.0008	−1.70857
			Indel	10	0.15%	0.00013	0.00031	−1.48424

Notes: π, average nucleotide differences per site between the two sequences; θ_w_, Watterson estimator; Tajima’s D, test for neutral selection (*: significant at p < 0.01; **: significant at p < 0.001; ***: significant at p < 0.0001)

Among the 10 soybean *FT* genes, *GmFT1a* had the most polymorphic sites (129), including 105 SNPs and 24 Indels (Table 1). However, because *GmFT1a* was one of the longest genes, only shorter than *GmFT6*, its nucleotide diversity (π) was 0.00521, lower than that of *GmFT1b* (π = 0.01102) and *GmFT2b* (π = 0.00588) (Table 1). *GmFT1b* had 93 polymorphic sites in the 2745 aligned bases, including 73 SNPs and 20 Indels (Table 1). *GmFT1b* nucleotide diversity (π) reached the peak of 0.01102 among the 10 soybean *FT* family genes, whereas *GmFT3a* had the lowest π of 0.00039, with only 11 polymorphic sites (Table 1). For the two polymorphic types, SNPs and Indels contributed differently to nucleotide diversity for each *FT* family gene with the contribution of the SNPs greater than that of Indels. *GmFT1a* was such a case, and SNP and Indel polymorphisms accounted for 0.00434 and 0.00087, respectively, with respect to the total nucleotide diversity (π). *GmFT1b* had a comparatively high π of SNPs of 0.00845; nevertheless, π of SNPs of *GmFT4* was much lower at 0.00009 (Table 1). *GmFT1b* also had the highest π of Indels, up to 0.00257, and the π of *GmFT2b* was slightly lower than that of *GmFT1b*, up to 0.00203, compared with the lowest π of the Indels of *GmFT3a* of only 0.00005 (Table 1). Figure 2 shows that *GmFT1b* had high polymorphisms, followed by *GmFT1a* and *GmFT2b* with polymorphisms lower than those of *GmFT1b*, whereas *GmFT3a* and *GmFT4* were conservative. The coding region of *GmFT4* was highly conserved, without any polymorphic sites, and *GmFT3a*, *GmFT5a* and *GmFT5b* were comparatively conservative, with only one polymorphic site in the coding region (Fig. 2 and Additional file 5: Table S5). To study the population selection pressure, we conducted neutral testing using Tajima’s D. Tajima’s D value of both *GmFT3b* and *GmFT5b* was positive, with their values reaching extremely significant levels (*p* < 0.001 and *p* < 0.0001), except that the SNPs of *GmFT3b* exhibited a significant level (*p* < 0.01) (Table 1). These data indicated that these regions of the two genes might experience balancing selection or population shrinkage.

**Fig. 3:**
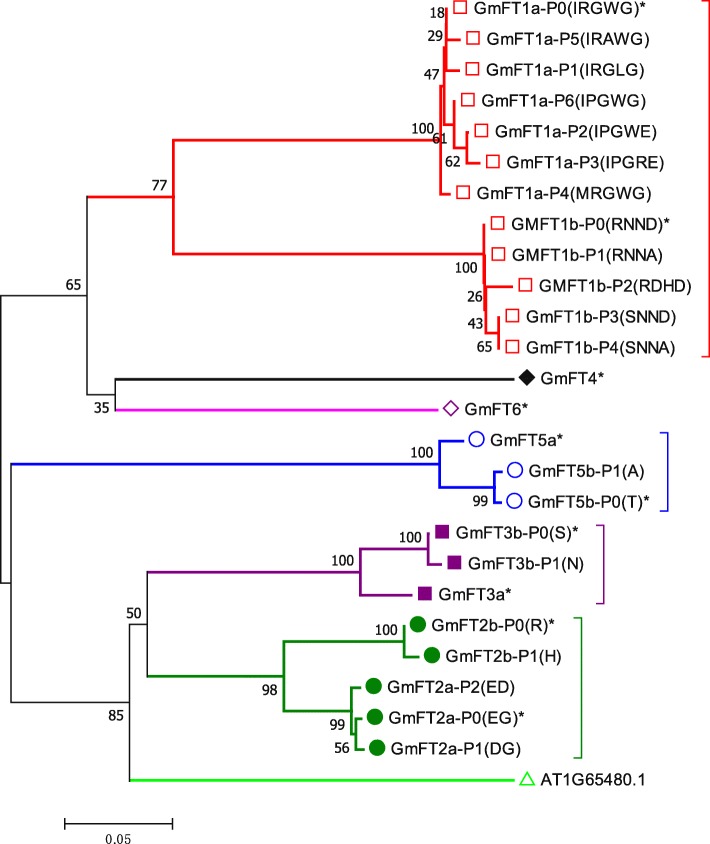
Phylogenetic relationships of different types of soybean FT proteins using the NJ method. Bootstrap percentage supports are indicated at the branches of the tree. ‘*’ represents the protein sequence type that is the same as that of Williams 82

In our study, we failed to identify any parsimony informative sites that led to premature stop codons in the coding region; however, some synonymous and nonsynonymous polymorphic sites existed. GmFT1a had seven types of protein, and GmFT1b had 5 types of protein, but GmFT3a, GmFT4, GmFT5a and GmFT6 had only one type of protein, indicating the high evolutionary conservation of these genes (Fig. 3). Compared with the polymorphic sites of the 10 soybean *FT* family genes in the Phytozome database, new polymorphic sites were found (Additional file 5: Table S5). For example, 94 new polymorphic sites were found in *GmFT1a* (Additional file 5: Table S5). These data suggested that the population we selected had extensive coverage of polymorphic sites and was particularly useful to research natural variations.

### Haplotype analysis of the 10 soybean *FT* family genes

**Fig. 4:**
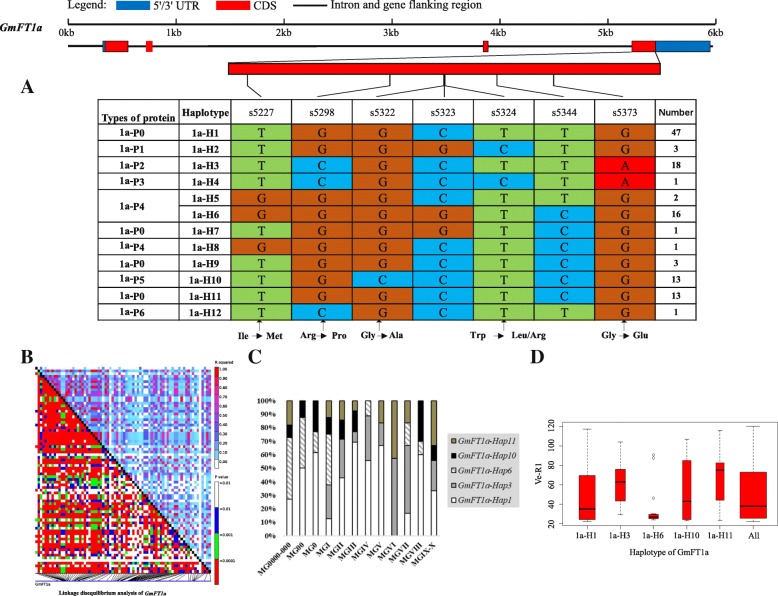
*GmFT1a* linkage disequilibrium analysis, haplotype definition, primary haplotypes distribution in different maturity groups and flowering time (Ve-R1) in the primary haplotypes*.* **a**: Haplotypes of *GmFT1a* region. The haplotype is shown as a linear combination of alleles. **b**: Linkage disequilibrium analysis of *GmFT1a*; **c**: Primary haplotypes distribution of *GmFT1a* in soybean varieties from different maturity groups; **d**: Flowering time (Ve-R1) of the primary haplotypes of *GmFT1a*. All means in the boxplot include all haplotypes of this gene. The width of the boxplot represents the sample size

**Fig. 5:**
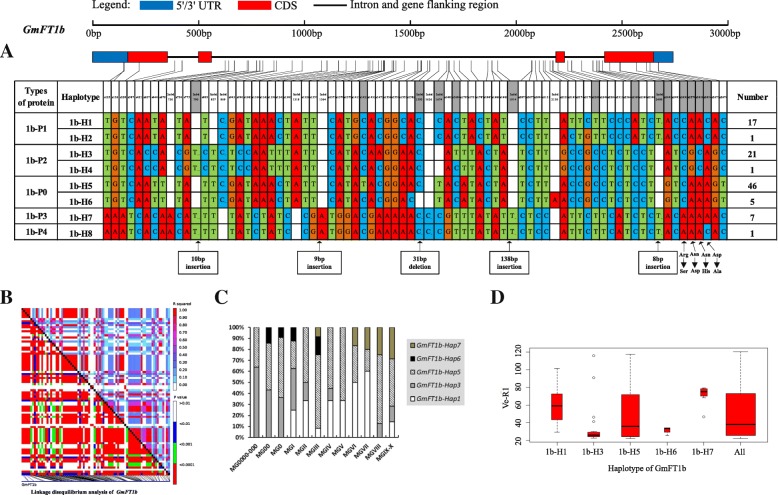
*GmFT1b* linkage disequilibrium analysis, haplotype definition, primary haplotypes distribution in different maturity groups and flowering time (Ve-R1) in the primary haplotypes*.* **a**: Haplotypes of *GmFT1b* region. The haplotype is shown as a linear combination of alleles. Site combination labeled with gray outline represents tagging haplotype (haplotype composition depends on fewer and critical variation sites); **b**: Linkage disequilibrium analysis of *GmFT1b*; C: Primary haplotypes distribution of *GmFT1b* in soybean varieties from different maturity groups; D: Flowering time (Ve-R1) of the primary haplotypes of *GmFT1b*. All means in the boxplot include all haplotypes of this gene. The width of the boxplot represents the sample size

Strong linkage disequilibrium (LD) has a large contribution to haplotype analysis and eases the identification of some SNPs and Indels in relation to phenotypes. Soybean *FT* family genes exhibited different levels of LD. Among the genes, *GmFT1a* and *GmFT1b* both had a similar level of polymorphism, and LD was weak in *GmFT1a* but strong in *GmFT1b* (Figs. 4 and 5). *GmFT3b* presented strong LD across the region from starting site to nearly the middle site, as a haplotype block, and across the entire region, *GmFT5b* presented some strong LD dispersed (Figs. 9 and 12). The entire gene region of *GmFT3a* could be defined as a haplotype block because of the strong LD of its polymorphic sites (Fig. 8).

**Fig. 6:**
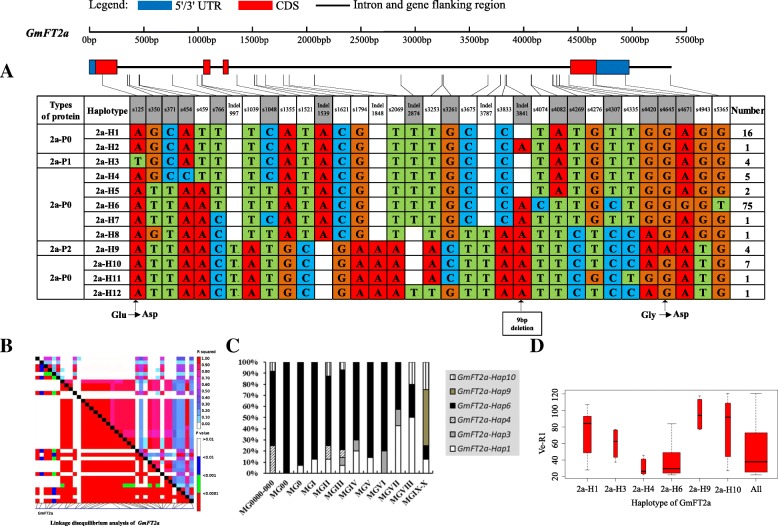
*GmFT2a* linkage disequilibrium analysis, haplotype definition, primary haplotypes distribution in different maturity groups and flowering time (Ve-R1) in the primary haplotypes*.* **a**: Haplotypes of *GmFT2a* region. The haplotype is shown as a linear combination of alleles. Site combination labeled with gray outline represents tagging haplotype (haplotype composition depends on fewer and critical variation sites); **b**: Linkage disequilibrium analysis of *GmFT2a*; **c**: Primary haplotypes distribution of *GmFT2a* in soybean varieties from different maturity groups; **d**: Flowering time (Ve-R1) of the primary haplotypes of *GmFT2a*. All means in the boxplot include all haplotypes of this gene. The width of the boxplot represents the sample size

**Fig. 7:**
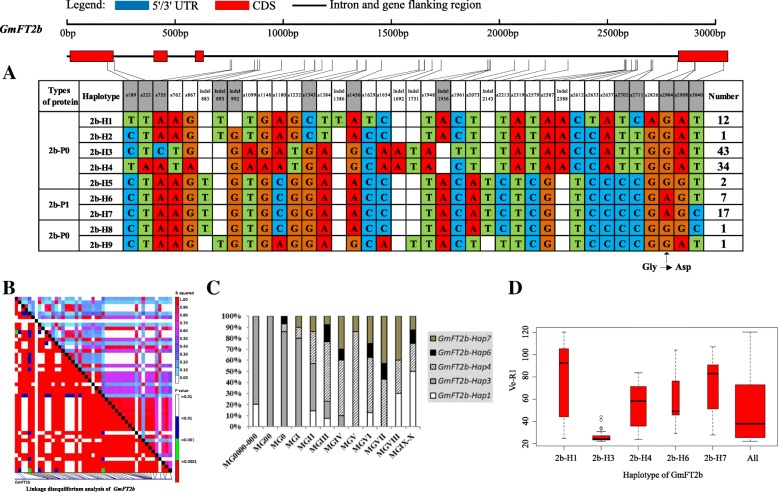
*GmFT2b* linkage disequilibrium analysis, haplotype definition, primary haplotypes distribution in different maturity groups and flowering time (Ve-R1) in the primary haplotypes*.* **a**: Haplotypes of *GmFT2b* region. The haplotype is shown as a linear combination of alleles. Site combination labeled with gray outline represents tagging haplotype (haplotype composition depends on fewer and critical variation sites); **b**: Linkage disequilibrium analysis of *GmFT2b*; **c**: Primary haplotypes distribution of *GmFT2b* in soybean varieties from different maturity groups; **d**: Flowering time (Ve-R1) of the primary haplotypes of *GmFT2b*. All means in the boxplot include all haplotypes of this gene. The width of the boxplot represents the sample size

**Fig. 8:**
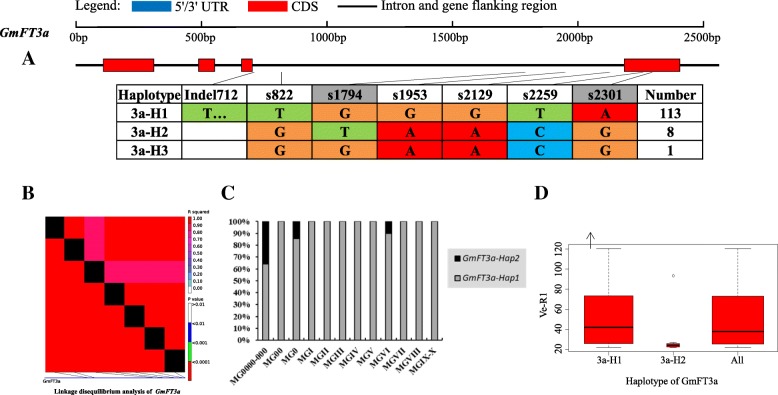
*GmFT3a* linkage disequilibrium analysis, haplotype definition, primary haplotypes distribution in different maturity groups and flowering time (Ve-R1) in the primary haplotypes*.* **a**: Haplotypes of *GmFT3a* region. The haplotype is shown as a linear combination of alleles. Site combination labeled with gray outline represents tagging haplotype (haplotype composition depends on fewer and critical variation sites); **b**: Linkage disequilibrium analysis of *GmFT3a*; **c**: Primary haplotypes distribution of *GmFT3a* in soybean varieties from different maturity groups; **d**: Flowering time (Ve-R1) of the primary haplotypes of *GmFT3a*. All means in the boxplot include all haplotypes of this gene. The width of the boxplot represents the sample size

**Fig. 9:**
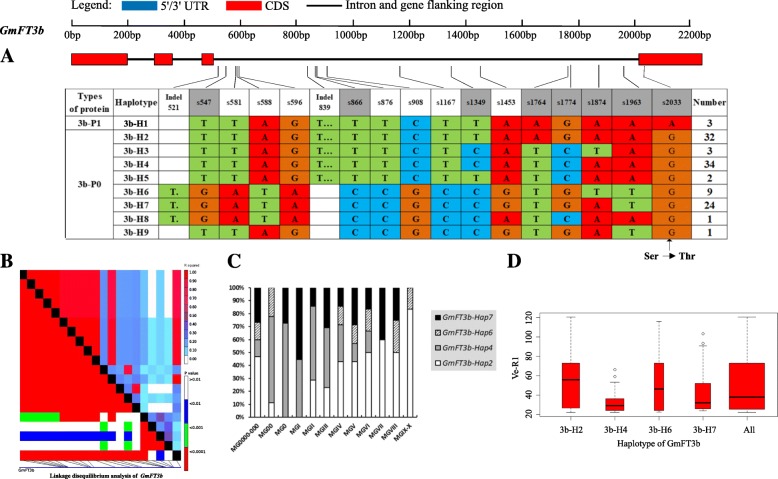
*GmFT3b* linkage disequilibrium analysis, haplotype definition, primary haplotypes distribution in different maturity groups and flowering time (Ve-R1) in the primary haplotypes*.* **a**: Haplotypes of *GmFT3b* region. The haplotype is shown as a linear combination of alleles. Site combination labeled with gray outline represents tagging haplotype (haplotype composition depends on fewer and critical variation sites); **b**: Linkage disequilibrium analysis of *GmFT3b*; **c**: Primary haplotypes distribution of *GmFT3b* in soybean varieties from different maturity groups; **d**: Flowering time (Ve-R1) of the primary haplotypes of *GmFT3b*. All means in the boxplot include all haplotypes of this gene. The width of the boxplot represents the sample size

**Fig. 10:**
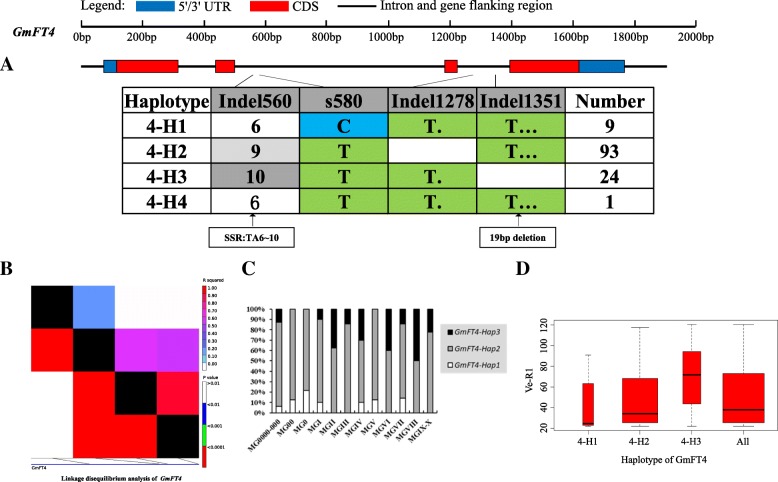
*GmFT4* linkage disequilibrium analysis, haplotype definition, primary haplotypes distribution in different maturity groups and flowering time (Ve-R1) in the primary haplotypes. **a**: Haplotypes of *GmFT4* region. The haplotype is shown as a linear combination of alleles. Site combination labeled with gray outline represents tagging haplotype (haplotype composition depends on fewer and critical variation sites); **b**: Linkage disequilibrium analysis of *GmFT4*; **c**: Primary haplotypes distribution of *GmFT4* in soybean varieties from different maturity groups; **d**: Flowering time (Ve-R1) of the primary haplotypes of *GmFT4*. All means in the boxplot include all haplotypes of this gene. The width of the boxplot represents the sample size

**Fig. 11:**
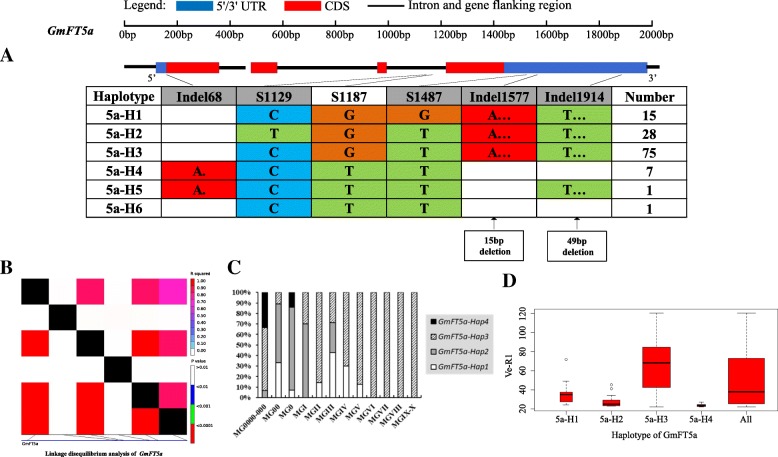
*GmFT5a* linkage disequilibrium analysis, haplotype definition, primary haplotypes distribution in different maturity groups and flowering time (Ve-R1) in the primary haplotypes*.* **a**: Haplotypes of *GmFT5a* region. The haplotype is shown as a linear combination of alleles. Site combination labeled with gray outline represents tagging haplotype (haplotype composition depends on fewer and critical variation sites); **b**: Linkage disequilibrium analysis of *GmFT5a*; **c**: Primary haplotypes distribution of *GmFT5a* in soybean varieties from different maturity groups; **d**: Flowering time (Ve-R1) of the primary haplotypes of *GmFT5a*. All means in the boxplot include all haplotypes of this gene. The width of the boxplot represents the sample size

**Fig. 12:**
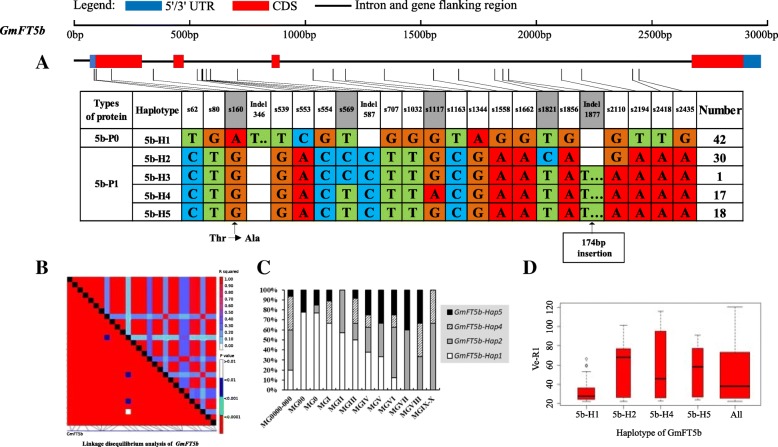
*GmFT5b* linkage disequilibrium analysis, haplotype definition, primary haplotypes distribution in different maturity groups and flowering time (Ve-R1) in the primary haplotypes*.* **a**: Haplotypes of *GmFT5b* region. The haplotype is shown as a linear combination of alleles. Site combination labeled with gray outline represents tagging haplotype (haplotype composition depends on fewer and critical variation sites); **b**: Linkage disequilibrium analysis of *GmFT5b*; **c**: Primary haplotypes distribution of *GmFT5b* in soybean varieties from different maturity groups; **d**: Flowering time (Ve-R1) of the primary haplotypes of *GmFT5b*. All means in the boxplot include all haplotypes of this gene. The width of the boxplot represents the sample size

**Fig. 13:**
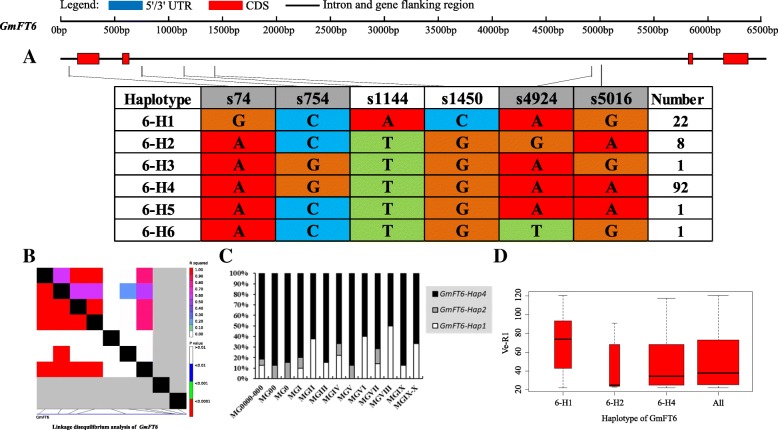
*GmFT6* linkage disequilibrium analysis, haplotype definition, primary haplotypes distribution in different maturity groups and flowering time (Ve-R1) in the primary haplotypes*.* **a**: Haplotypes of *GmFT6* region. The haplotype is shown as a linear combination of alleles. Site combination labeled with gray outline represents tagging haplotype (haplotype composition depends on fewer and critical variation sites); **b**: Linkage disequilibrium analysis of *GmFT6*; **c**: Primary haplotypes distribution of *GmFT6* in soybean varieties from different maturity groups; **d**: Flowering time (Ve-R1) of the primary haplotypes of *GmFT6*. All means in the boxplot include all haplotypes of this gene. The width of the boxplot represents the sample size

On the basis of sequence alignment and LD, haplotype analysis proceeded for the 10 soybean *FT* family genes in the 127 varieties used in this study. Haplotypes of the 10 soybean *FT* family genes, variation types and polymorphic sites used for composing the haplotype are listed in Additional file 7: Table S7, Additional file 8: Table S8 and Additional file 9: Table S9. These results indicated that 1a-H3, 1b-H1, 1b-H6, 1b-H7, 2a-H1, 2a-H3, 2a-H4, 2a-H9, 2b-H3, 2b-H4, 2b-H6, 2b-H7, 3b-H4, 5a-H1, 5a-H2, 5a-H4 and 5b-H1 showed some association with flowering time and maturity, whereas the haplotypes of *GmFT3a*, *GmFT4* and *GmFT6* did not exhibit any apparent associations (Figs. 4, 5, 6, 7, 8, 9, 10, 11, 12, 13). Haplotype combinations of soybean *FT* family genes showed some associations, i.e., 1a-H3 appeared in the varieties with the haplotype of 1b-H1, and 2a-H1 and 2a-H3 appeared in the varieties with 2b-H6 and 2b-H7, respectively, and 3b-H4 appeared in the varieties with 5b-H1 (Additional file 7: Table S7). A total of 8 haplotypes of *GmFT1b*, with 64 SNPs and 12 Indels, were categorized in 99 varieties (Fig. 5 and Additional file 9: Table S9), whereas a total of 4 haplotypes of *GmFT4* were defined in 127 varieties with 1 SNP and 3 Indels because of its sequence conservation (Fig. 10). 1b-H5 was the most abundant, accounting for 46 varieties; 1b-H1 appeared in varieties in MGI and late-maturing groups, and the Ve-R1 of 1b-H1 varied from 29.2 to 101.1 days; 1b-H7 was distributed in varieties in MGIII and late-maturing groups, and the Ve-R1 of 1b-H7 ranged from 46.6 to 79.4 days, later flowering than that of early-maturing varieties; 1b-H6 appeared in varieties in the maturity groups between MG00 and MGIII, with early flowering, and its flowering time ranged from 25.3 to 34.3 days (Fig. 5, Additional file 6: Table S6 and Additional file 7: Table S7). 1b-P1, a type of GmFT1b protein sequence, appeared in the varieties of 1b-H1 and 1b-H2. The latter was only found in Jindou 39 belonging to MGIV. 1b-P3 appeared in the varieties of 1b-H7 (Fig. 5, Additional file 7: Table S7 and Additional file 10: Table S10). Six haplotypes of *GmFT5a* were defined in 127 varieties (Fig. 11 and Additional file 9: Table S9). 5a-H3 was the most abundant, accounting for 75 varieties, more than half of the population; 5a-H1 was distributed in varieties with their maturity groups between MG00 and MGVI, and the Ve-R1 of 5a-H1 varied from 24.3 to 49.3 days, except that Diandou 7 belonging to MGV flowered 71.7 days after emergence; 5a-H2 was distributed in varieties with the maturity group MGIII and early-maturing groups, and the Ve-R1 of 5a-H2 varied from 23.1 to 45.6 days; 5a-H4 was distributed in varieties with their maturity groups belonging to MG0000, MG000 and MG00, and the Ve-R1 of 5a-H4 ranged from 22.2 to 27.4 days, flowering comparatively early (Fig. 11, Additional file 6: Table S6 and Additional file 7: Table S7).

By analyzing the LD of polymorphic sites, a small and critical quantity of SNPs and Indels are selected and can define haplotypes, known as tagging haplotype [46]. According to LD analysis and polymorphic site features, some SNPs and Indels were chosen to form tagging haplotype; for example, only 14 polymorphic sites represented the *GmFT1b* haplotype (Fig. 5), and only five polymorphic sites were used for the *GmFT5b* tagging haplotype (Fig. 12), whereas the haplotype definition of *GmFT1b* and *GmFT5b* contained 76 and 23 polymorphic sites, respectively (Figs. 5 and 12 and Additional file 9: Table S9). Similarly, we only required 16, 13, 2, and 8 polymorphic sites to define tagging haplotype for *GmFT2a*, *GmFT2b*, *GmFT3a* and *GmFT3b*, respectively, but the haplotype definition of them contained 34, 39, 7, and 17 polymorphic sites, respectively (Figs. 6, 7, 8, 9 and Additional file 9: Table S9).

### Polymorphic sites associated with flowering time

GLM analysis showed that some polymorphic sites were associated with flowering time among *GmFT1b, GmFT2a, GmFT2b, GmFT5a,* and *GmFT5b*, which are listed in Additional file 4: Table S4. Fourteen polymorphic sites were in 1b-H1 and 1b-H7 collectively, and they showed a relationship with flowering time at a significance level of *p* < 0.01 (Additional file 4: Table S4). Among the sites, an 8 bp fragment inserted in Intron3, namely, Indel2409, was only 4 bp distant from exon4 (Additional file 4: Table S4). s4645 in exon4 appeared in 2a-H9 and showed a relationship with flowering time at a significance level of p < 0.01, leading to G to D at aa169 (Additional file 10: Table S10 and Additional file 4: Table S4). s755 and s1961 in 2b-H3 and s2904 in 2b-H6 and 2b-H7 showed a relationship with flowering time at a significance level of p < 0.01, and s2904 in exon4 resulted in nonsynonymous substitution, R to H at aa126 (Additional file 10: Table S10 and Additional file 4: Table S4). Two polymorphic sites, s1129 in 5a-H2 and Indel1914 in 5a-H4 and 5a-H6, as a 49 bp fragment deleted in the 3’UTR, showed a relationship with flowering time at a significance level of p < 0.01 (Additional file 4: Table S4). Eighteen polymorphic sites in 5b-H1, with strong LD among them, showed a relationship with flowering time at a significance level of p < 0.01 of which s160 in exon1 led to T to A at aa27 (Additional file 10: Table S10 and Additional file 4: Table S4). The above-mentioned polymorphic sites may have some effects on flowering time and maturity through regulating the transcription of soybean *FT* family genes or only act as markers associated with those traits.

## Discussion

### The 10 soybean *FT* family genes exhibited variations in sequence polymorphism

The flowering time and maturity of the varieties ranged from 22.2 to 120.5 days and from 61.4 to longer than 155.7 days, respectively, indicating a high diversity in these parameters (Additional file 6: Table S6). This finding showed that the soybean population in our study was appropriate for the study of genetic factors influencing flowering time and maturity in soybeans.

Ten *FT* family genes are in the soybean genome [26, 30]. Only a few of the genes have been studied and their function identified in soybean [22, 23, 26, 30, 31, 35]. Liu et al. found *GmFT1a* functions divergently to delay flowering and maturing, a function significantly different from that of two known flowering promoters *GmFT2a* and *GmFT5a* [31]. Therefore, studying their polymorphisms will help us to learn the evolutionary trends and breeding selection effects on the genes. Based on the Sanger sequencing results, the *FT* genes exhibited variations in all 127 varieties in the aspect of sequence polymorphism. However, the genes had different levels of polymorphism, and *GmFT1b, GmFT1a* and *GmFT2b* had the highest levels of genetic variation, whereas *GmFT3a* and *GmFT4* were highly conserved. Consistently, in the relatively conserved coding region, *GmFT1b*, *GmFT1a* and *GmFT2b* had more polymorphisms than those of *GmFT3a* and *GmFT4*. Jiang et al. did not find any variations in the coding region of *GmFT2a* [34], and only a synonymous site has been found from the 31-Soybean Genome Resequence project [49]. In the current study, two nonsynonymous variations were found in the coding region of *GmFT2a* suggesting that the selected varieties had extensive genetic variation.

### *GmFT3b* and *GmFT5b* may have experienced breeding selection in soybean domestication and breeding

Zhu et al. found that soybean nucleotide diversity was much lower than that in Arabidopsis and maize, with the value of θ_w_ only 0.00097, in studying sequence polymorphism of the soybean genome [50]. The θ_w_ values of the 10 soybean *FT* family genes ranged from 0.00029–0.00569 in the aspect of SNPs in this study. *GmFT3a* (θ_w_ = 0.00073), *GmFT4* (θ_w_ = 0.00029), *GmFT5a* (θ_w_ = 0.00084) and *GmFT6* (θ_w_ = 0.0008) should be conserved after strong selection. *GmFT1a* (θ_w_ = 0.0035), *GmFT1b* (θ_w_ = 0.00569), *GmFT2a* (θ_w_ = 0.00133), *GmFT2b* (θ_w_ = 0.00253), *GmFT3b* (θ_w_ = 0.00169) and *GmFT1b* (θ_w_ = 0.00144) might have experienced weak selection pressure and had high polymorphism in long-term evolution. This finding also reflected that the selected population had a wide genetic base. Because the ten *FT* family genes are diverse in the control of flowering and maturity, these genes could play concerto roles to elaborately regulate flowering and maturing and help soybean to adapt to greatly diverse environments and breeding selections. Neutral test indicated that *GmFT3b* and *GmFT5b* had some selected signals and might have experienced breeding selection. The polymorphic sites of *GmFT3b* and *GmFT5b* had a high proportion of strong LD (Figs. 9 and 12), which suggested that *GmFT3b* and *GmFT5b* underwent relevant selection in adaptation to different environments, consistent with Tajima’s D test.

### Haplotypes of distinct soybean *FT* family genes and their combinations

Haplotype-based analysis is more informative than SNP-based analysis and is more powerful in analyzing the association with phenotypes [51]. By conducting haplotype analysis of the 10 soybean *FT* family genes, we found that seven *FT* family genes, including *GmFT1a*, *GmFT1b*, *GmFT2a*, *GmFT2b*, *GmFT3b*, *GmFT5a*, and *GmFT5b*, had some haplotypes associated with flowering time and maturity. However, the other genes, *GmFT3a, GmFT4* and *GmFT6*, did not exhibit any relations with flowering time and maturity, which could be because the three genes were relatively conservative. 1b-H7 appeared in varieties in the MGIII and late-maturity groups, and its protein sequence type was 1b-P3 (Fig. 5). A 138 bp fragment inserted in Intron2 appeared in 1b-H7 (Fig. 5). We observed that 2a-H9 was distributed in the varieties with the maturity groups of MGIX and MGX, and 5a-H4 appeared in the varieties with the maturity groups of MG0000, MG000 and MG00 (Figs. 6 and 11). The flowering time of soybean with the haplotypes of 2a-H9 and 5a-H4 ranged from 76.9 to 117.6 days and from 22.2 to 27.4 days, respectively (Figs. 6 and 11). These haplotypes will be helpful in studying soybean *FT* family genes and can be used as markers associated with flowering time and maturity.

Haplotype combinations of soybean *FT* family genes had some relations. 1a-H3 and 1b-H1, 2a-H1 and 2b-H6, 2a-H3 and 2b-H7, and 3b-H4 and 5b-H1 were the combinations. These haplotype combinations were mostly in the same varieties. According to their location in the genome and phylogenetic relationships, *GmFT1a* and *GmFT1b*, which are located in Chr18, had the closest evolutionary relationship (1011 kb apart); *GmFT2a* and *GmFT2b*, which are located in Chr16, had the closest evolutionary relationship (34 kb apart); and *GmFT3b* and *GmFT5b*, which are located in Chr19, were only 16 kb apart. In the long process of natural selection and domestication, these short distances might result in linkage disequilibrium. As a result of certain selection effects, some polymorphic sites might have been inherited together in the adaptation to some environments.

### Polymorphisms of soybean *FT* family genes have relations to flowering time

We chose polymorphic sites that only existed in the haplotypes with relations to flowering time and maturity. Further association analysis was conducted with GLM, and 38 polymorphic sites showed a significant association with flowering time at the level of *p* < 0.01. These polymorphic sites may be subjected to natural and artificial selection in adaptation to diverse environments and have effects on pre- and postflowering photoperiod responses. An 8 bp fragment insertion in Intron3, close to exon4, appeared in 1b-H1 and 1b-H7 collectively and might have an effect on flowering time. This insertion might regulate *GmFT1b* expression directly or indirectly through changing the alternative splicing of *GmFT1b. GmFT2a* and *GmFT5a* coordinately control flowering and enable the adaptation of soybean to a wide range of photoperiodic environments [26]. A nonsynonymous site in exon4 of *GmFT2a*, namely, s4645, appeared in 2a-H9 and was distributed in the varieties with their maturity groups belonging to MGIX and MGX. The change at the site resulted in an amino acid substitution from nonpolar G_169_ to acidic D_169_. Notably, the flowering inhibitor *GmFT1a* has an amino acid of D_169_ in the same location, indicating that this amino acid substitution might have an important role in the function of *GmFT2a*. A 49 bp fragment deleted in the 3′ UTR appeared in 5a-H4 and 5a-H6 and was distributed in the varieties of MG0000, MG000 and MG00. They may affect the function of *GmFT2a* and *GmFT5a* in regulating soybean flowering.

## Conclusions

Soybean varieties from the earliest maturing MG0000 to the latest maturing MGX showed high diversity in flowering time and maturity. The 10 soybean *FT* family genes exhibited variation in the aspect of genome sequence polymorphism. *GmFT3b* and *GmFT5b* had some selected signals and might have experienced breeding selection in the process of natural selection and domestication. By analyzing the haplotypes, we found that 1a-H3, 1b-H1, 1b-H6, 1b-H7, 2a-H1, 2a-H3, 2a-H4, 2a-H9, 2b-H3, 2b-H4, 2b-H6, 2b-H7, 3b-H4, 5a-H1, 5a-H2, 5a-H4 and 5b-H1 showed correlative relationships with flowering time and maturity; whereas *GmFT3a*, *GmFT4* and *GmFT6* did not have any apparent connections. Thirty-eight polymorphic sites showed a significant association with flowering time at the level of p < 0.01.

## Additional files

Additional file 1: **Table S1**. Varieties and their respective maturity group and origin. (DOCX 17 kb)

Additional file 2: **Table S2**. Sequence of primers for the 10 *FT* family genes in soybean. (DOCX 15 kb)

Additional file 3: **Table S3**. PCR reaction conditions for different templates of the *FT* family genes in soybean. (DOCX 13 kb)

Additional file 4: **Table S4**. General linear model association of polymorphic sites of soybean *FT* family genes with relation to flowering time and growth duration. (DOCX 23 kb)

Additional file 5: **Table S5**. Polymorphic sites of the 10 soybean *FT* family genes from the present study. (DOCX 56 kb)

Additional file 6: **Table S6**. Flowering time (Ve-R1) and growth duration (Ve-R7 and Ve-R8) data in Beijing, China, in 2015. (DOCX 21 kb)

Additional file 7: **Table S7**. Haplotypes of the 10 soybean *FT* family genes in 127 varieties covering 14 maturity groups. (DOCX 38 kb)

Additional file 8: **Table S8**. Polymorphic sites used for defining the haplotypes of the 10 soybean *FT* family genes. (DOCX 14 kb)

Additional file 9: **Table S9**. Polymorphic site features used for defining the haplotypes of the 10 soybean *FT* family genes. (DOCX 16 kb)

Additional file 10: **Table S10**. Different types of soybean FT proteins and their haplotype distribution. (DOCX 18 kb)

## Abbreviations

CTAB: Cetyl trimethyl ammonium bromide
FT: Flowering Locus T
GLM: General linear model
LD: Linkage disequilibrium
MG: Maturity group
NJ: Neighbor-joining
PCR: Polymerase chain reaction
SNP: Single nucleotide polymorphism
