# Supplementary material for: Natural variations of FT family genes in soybean varieties covering a wide range of maturity groups
Source: BMC Genomics. 2019 Mar 20;20:230. doi: 10.1186/s12864-019-5577-5 (PMC6425728; doi:10.1186/s12864-019-5577-5)
Supplement: Supplementary file 1 — Table S1. Varieties and their respective maturity group and origin. (DOCX 17 kb) [file 12864_2019_5577_MOESM1_ESM.docx]

**Table S1. Varieties and their respective maturity group and origin**

| **Variety** | **Maturity group** | **Origin** | **Variety** | **Maturity group** | **Origin** | **Variety** | **Maturity group** | **Origin** |
| --- | --- | --- | --- | --- | --- | --- | --- | --- |
| Star4/75 | 0000 | Russia | Taixingheidou | I | China | TN04-5321 | V | USA |
| R-4 | 0000 | Russia | Haroson | I | Canada | Shangdou 14 | V | China |
| Hujiao07-2479 | 000 | China | Tiefeng 19 | I | China | Diandou 7 | V | China |
| Paula | 000 | Russia | Parker | I | USA | Desha | VI | USA |
| Linbei 8 | 000 | China | Granite | I | USA | Boggs | VI | USA |
| Maple Presto | 000 | Canada | NE1900 | I | USA | Suxiandou 19 | VI | China |
| Hujiao07-2123 | 000 | China | Jilin 20 | II | China | G01-PR16 | VI | USA |
| R2 | 000 | Russia | Holt | II | USA | Zhongdou 38 | VI | China |
| Rassvet | 000 | Russia | Century 84 | II | USA | Musen | VI | USA |
| Heihe 35 | 000 | China | Olympus | II | USA | D95-6271 | VI | USA |
| OAC Vision | 000 | Canada | LN92-7369 | II | USA | Wuhuasiyuehuang | VI | China |
| Dongnong41-C | 000 | China | IL1 | II | USA | Nannong 493/1 | VI | China |
| Jug 30 | 000 | Russia | Yongchengzihuadou | II | China | Hengyangbayueqing | VI | China |
| Mageva | 000 | Russia | Xiangchundou 24 | II | China | Benning | VII | USA |
| Dongnong36 | 000 | China | Tiefeng 33 | III | China | Santee | VII | USA |
| Dongnong41 | 000 | China | Tiefeng 31 | III | China | Stonewall | VII | USA |
| Beidou 16 | 00 | China | Jindou 19 | III | China | Hagood | VII | USA |
| Mengdou 11 | 00 | China | Athow | III | USA | Nanxiadou 25 | VII | China |
| Dongnong 44 | 00 | China | Zhonghuang30 | III | China | Tongshanbaopihuang | VII | China |
| McCall | 00 | USA | KS3494 | III | USA | Huangfengwo | VII | China |
| Mengdou 32 | 00 | China | LN89-5699 | III | USA | Dowling | VIII | USA |
| Maple Ridge | 00 | Canada | Williams 82 | III | USA | Motte | VIII | USA |
| Daksoy | 00 | USA | Xudou 9 | III | China | Prichard | VIII | USA |
| Agassiz | 00 | USA | Zhongdou 39 | III | China | Aijiaoqing | VIII | China |
| Canatto | 00 | Canada | Zhonghuang13 | III | China | Nandou 12 | VIII | China |
| Heihe 18 | 0 | China | Huaidou 9 | III | China | Lanxidaqingdou | VIII | China |
| Heihe 43 | 0 | China | IL2 | III | USA | Shangraodaqingsi | VIII | China |
| Hefeng 25 | 0 | China | Huachun 6 | III | China | Nandou 17 | VIII | China |
| Heihe 27 | 0 | China | Flyer | IV | USA | Guixia 3 | VIII | China |
| Dengke 1 | 0 | China | Omaha | IV | USA | Pinguohuangdou | VIII | China |
| Beidou 37 | 0 | China | CF461 | IV | USA | Qiudou 1 | VIII | China |
| Traill | 0 | USA | Zheng 92116 | IV | China | Zigongdongdou | VIII | China |
| Fengshou 12 | 0 | China | Jindou 39 | IV | China | 'IAC-8 | IX | Brazil |
| Dongnong 4 | 0 | China | Calhoun | IV | USA | 'UFV-3 | IX | Brazil |
| Norpro | 0 | USA | Shanning 16 | IV | China | 'FT-15 | IX | Brazil |
| Jiangmodou 1 | 0 | China | Guandou 2 | IV | China | 'Alamo | IX | USA |
| Barnes | 0 | USA | UA 4805 | IV | USA | I.C. 192 | IX | India |
| Dawson | 0 | USA | Houzimao | IV | China | Jupiter | IX | USA |
| Chico | 0 | USA | Nathan | V | USA | CIGRAS-06 | X | Costa Rica |
| Heinong 16 | I | China | Holladay | V | USA | CIGRAS-51 | X | Costa Rica |
| Heinong 26 | I | China | Hutcheson | V | USA | Jiangledaqingdou | X | China |
| Suinong 14 | I | China | R01-3474F | V | USA |  |  |  |
| Kato | I | USA | Dian 86-4 | V | China |  |  |  |
