# Supplementary material for: Natural variations of FT family genes in soybean varieties covering a wide range of maturity groups
Source: BMC Genomics. 2019 Mar 20;20:230. doi: 10.1186/s12864-019-5577-5 (PMC6425728; doi:10.1186/s12864-019-5577-5)
Supplement: Supplementary file 2 — Table S2. Sequence of primers for the 10 FT family genes in soybean. (DOCX 15 kb) [file 12864_2019_5577_MOESM2_ESM.docx]

**Table S2. Sequence of primers for the 10 *FT* family genes in soybean**

| **Gene** | **Template** | **Primer** | **Sequence (5'→3')** | **Position in genome** |
| --- | --- | --- | --- | --- |
| *GmFT1a* | GmFT1as | GmFT1as-1F | TTGTAGAGGATGAAGTCAAAATAT | 57653318...57653341 |
|  | GmFT1as | GmFT1as-1R | ACAAAACTGATACCAACTAAACAT | 57655723...57655746 |
|  | GmFT1am | GmFT1am-1F | TAAAACTCCCACGGTTACACAATA | 57655353...57655376 |
|  | GmFT1am | GmFT1am-1R | GATCAGACATAATAACACGAAGGC | 57657265...57657288 |
|  | GmFT1ah | GmFT1ah-1F | TATTGGACGCCACTTTGCTTAAAT | 57657072...57657095 |
|  | GmFT1ah | GmFT1ah-1R | TCCTGACTTCTTTCACGTTTTTGT | 57659298...57659321 |
| *GmFT1b* | GmFT1b | GmFT1b-2F | TCTATGCAAGTATGAAAGGGATAT | 57670363...57670386 |
|  | GmFT1b | GmFT1b-2R | TTAAAGTAAACCCAACAATTTGAG | 57673010...57673033 |
| *GmFT2a* | GmFT2aq | GmFT2aq-1F | CAATGGAATCGAGGCTATTGACTA | 31109921...31109944 |
|  | GmFT2aq | GmFT2aq-1R | CAGGTTTGCTGATCGTACTTTACA | 31112565...31112588 |
|  | GmFT2am | GmFT2am-1F | TGTGGACTGTGTTTTCCATTGAC | 31112346…31112368 |
|  | GmFT2am | GmFT2am-1R | ATGGTCCTACTACTGCTGTCC | 31114151...31114171 |
|  | GmFT2ae | GmFT2ae-1F | TATGTATGAGTGTTTCTGGCTTTC | 31113894...31113917 |
|  | GmFT2ae | GmFT2ae-1R | ATGACTTTGAGGTTATATTTGGTT | 31115422...31115445 |
| *GmFT2b* | GmFT2b | GmFT2b-1F | TAGTTGGTGTCATAACAAAGCATA | 31148761...31148784 |
|  | GmFT2b | GmFT2b-1R | CACATTCAAAAGTCCCTTATTTTA | 31151874...31151897 |
| *GmFT3a* | GmFT3aq | GmFT3aq-1F | CTCACGCGTGCTTTCAATACATAA | 4165016...4164993 |
|  | GmFT3aq | GmFT3aq-1R | ATTTACATTTTCACGCTCATTCACT | 4163450…4163426 |
|  | GmFT3ah | GmFT3ah-1F | AAATTACCCAGACCGGGACAAACA | 4163781...4163758 |
|  | GmFT3ah | GmFT3ah-1R | TTGACAAACTCCATGATCCCAAAG | 4162282...4162259 |
| *GmFT3b* | GmFT3b | GmFT3b-2F | TGGGTGTTGTAGTACAATTGTTTG | 36030550...36030573 |
|  | GmFT3b | GmFT3b-2R | GATTACCTTTCTTATTTGGGTGCT | 36032928...36032951 |
| *GmFT4* | GmFT4 | GmFT4-1F | ATCTTAGAGGAAAAGGAAAACCAGAAT | 47459967...47459941 |
|  | GmFT4 | GmFT4-1R | GGAATTTACTTACCGTTGAGACCATAT | 47457943...47457917 |
| *GmFT5a* | GmFT5a | GmFT5a-3F | GAGAAAAGAAAACATTTCATCGTACAACA | 4137921...4137893 |
|  | GmFT5a | GmFT5a-3R | TACAAACAATAGCAAAGCACATATTCAAAC | 4135752...4135723 |
| *GmFT5b* | GmFT5b | GmFT5b-1F | ACCAAGGGAAAGAAAAGTGGTACT | 36048867…36048890 |
|  | GmFT5b | GmFT5b-1R | CATCAGATCAAAGGGCATAGACAT | 36051859…36051882 |
| *GmFT6* | GmFT6s | GmFT6s-1F | ATACACCTTGCAACTTTGGGTATG | 47473595…47473572 |
|  | GmFT6s | GmFT6s-1R | AAATGTTCCAGAAGTTGAACAATGAT | 47471324…47471299 |
|  | GmFT6m | GmFT6m-1F | TACTTTCATCTTAACCATTCATCT | 47471702…47471679 |
|  | GmFT6m | GmFT6m-1R | ACATCCTTAGTCCAATAACTTCAA | 47469204…47469181 |
|  | GmFT6e | GmFT6e-1F | TTATCTATTTATTGGGGTTGAGGA | 47469462…47469439 |
|  | GmFT6e | GmFT6e-1R | TCATTCTTGGCAACTAAAACACTT | 47466994...47466971 |
