# Supplementary material for: Natural variations of FT family genes in soybean varieties covering a wide range of maturity groups
Source: BMC Genomics. 2019 Mar 20;20:230. doi: 10.1186/s12864-019-5577-5 (PMC6425728; doi:10.1186/s12864-019-5577-5)
Supplement: Supplementary file 3 — Table S3. PCR reaction conditions for different templates of the FT family genes in soybean. (DOCX 13 kb) [file 12864_2019_5577_MOESM3_ESM.docx]

**Table S3. PCR reaction conditions for different templates of the *FT* family genes in soybean**

| **Template** | **Product length (bp)** | **DNA Polymerase** | **PCR reaction conditions of different templates** |
| --- | --- | --- | --- |
| GmFT1as | 2429 | KOD-FX | 94ºC 2 min,(98ºC 10 s,57ºC 30 s,68ºC 2 min 35 s)cycle 30,68ºC 5min |
| GmFT1am | 1936 | KOD-FX | 94ºC 2 min,(94ºC 15 s,60ºC 30 s,68ºC 2 min)cycle 30,68ºC 5min |
| GmFT1ah | 2250 | KOD-Plus-Neo | 94ºC 2 min,(98ºC 10 s,58ºC 30 s,68ºC 1 min 15 s)cycle 30,68ºC 5min |
| GmFT1b | 2671 | KOD-Plus-Neo | 94ºC 2 min,(98ºC 10 s,59ºC 30 s,68ºC 1 min 25 s)cycle 30,68ºC 5min |
| GmFT2aq | 2668 | KOD-Plus-Neo | 94ºC 2 min,(98ºC 10 s,58ºC 30 s,68ºC 1 min 25 s)cycle 30,68ºC 5min |
| GmFT2am | 1826 | KOD-FX | 94ºC 2 min,(94ºC 15 s,57ºC 30 s,68ºC 2 min)cycle 30,68ºC 5min |
| GmFT2ae | 1552 | KOD-Plus-Neo | 94ºC 2 min,(98ºC 10 s,53ºC 30 s,68ºC 50 s)cycle 30,68ºC 5min |
| GmFT2b | 3137 | KOD-Plus-Neo | 94ºC 2 min,(98ºC 10 s,58ºC 30 s,68ºC 1 min 45 s)cycle 30,68ºC 5min |
| GmFT3aq | 1591 | KOD-Plus-Neo | 94ºC 2 min,(98ºC 10 s,59ºC 30 s,68ºC 50 s)cycle 35,68ºC 5min |
| GmFT3ah | 1523 | KOD-Plus-Neo | 94ºC 2 min,(98ºC 10 s,60ºC 30 s,68ºC 50 s)cycle 30,68ºC 5min |
| GmFT3b | 2402 | KOD-Plus-Neo, KOD-FX | 94ºC 2 min,(98ºC 10 s,53ºC 30 s,68ºC 1 min 25 s)cycle 30,68ºC 5min  94ºC 2 min,(94ºC 15 s,55ºC 30 s,68ºC 2 min 30 s)cycle 30,68ºC 5min |
| GmFT4 | 2051 | KOD-Plus-Neo | 94ºC 2 min,(98ºC 10 s,53ºC 30 s,68ºC 1 min 15 s)cycle 30,68ºC 5min |
| GmFT5a | 2199 | KOD-Plus-Neo | 94ºC 2 min,(98ºC 10 s,55ºC 30 s,68ºC 1 min 10 s)cycle 30,68ºC 5min |
| GmFT5b | 3016 | KOD-Plus-Neo, KOD-FX | 94ºC 2 min,(98ºC 10 s,55ºC 30 s,68ºC 1 min 45 s)cycle 30,68ºC 5min  94ºC 2 min,(98ºC 10 s,57ºC 30 s,68ºC 3 min 5 s)cycle 30,68ºC 5min |
| GmFT6s | 2297 | KOD-FX | 94ºC 2 min,(94ºC 15 s,58ºC 30 s,68ºC 2 min 10 s)cycle 30,68ºC 5min |
| GmFT6m | 2522 | KOD-FX | 94ºC 2 min,(98ºC 10 s,55ºC 30 s,68ºC 2 min 35 s)cycle 30,68ºC 5min |
| GmFT6e | 2492 | KOD-FX | 94ºC 2 min,(98ºC 10 s,55ºC 30 s,68ºC 2 min 35 s)cycle 30,68ºC 5min |
