# Supplementary material for: Natural variations of FT family genes in soybean varieties covering a wide range of maturity groups
Source: BMC Genomics. 2019 Mar 20;20:230. doi: 10.1186/s12864-019-5577-5 (PMC6425728; doi:10.1186/s12864-019-5577-5)
Supplement: Supplementary file 4 — Table S4. General linear model association of polymorphic sites of soybean FT family genes with relation to flowering time and growth duration. (DOCX 23 kb) [file 12864_2019_5577_MOESM4_ESM.docx]

**Table S4. General linear model association of polymorphic sites of soybean *FT* family genes with relation to flowering time and growth duration**

|  | **Trait** | **Ve-R1** | |  | **Trait** | **Ve-R1** | |
| --- | --- | --- | --- | --- | --- | --- | --- |
| **Gene** |  | **p-value** | **R^2^** | **Gene** |  | **p-value** | **R^2^** |
| *GmFT1a* | Indel1567 | 0.0495 | 0.0326 | *GmFT1b* | s2111 | 0.0212 | 0.0536 |
|  | s3389 | 0.2244 | 0.0126 |  | Indel2130 | 0.1354 | 0.0228 |
|  | s4038 | 0.0495 | 0.0326 |  | s2174 | 0.0042 | 0.0815 |
|  | s4501 | 0.0495 | 0.0326 |  | s2200 | 0.0042 | 0.0815 |
|  | s5298 | 0.0366 | 0.0368 |  | s2248 | 0.0042 | 0.0815 |
|  | s5373 | 0.0527 | 0.0317 |  | s2264 | 0.0042 | 0.0815 |
| *GmFT1b* | s125 | 0.0212 | 0.0536 |  | s2311 | 0.0212 | 0.0536 |
|  | s126 | 0.0212 | 0.0536 |  | s2364 | 0.1086 | 0.0263 |
|  | s220 | 0.0212 | 0.0536 |  | s2380 | 0.1086 | 0.0263 |
|  | s387 | 0.0212 | 0.0536 |  | s2388 | 0.0042 | 0.0815 |
|  | s421 | 0.0212 | 0.0536 |  | s2396 | 0.0042 | 0.0815 |
|  | Indel730 | 0.0212 | 0.0536 |  | Indel2409 | 0.0042 | 0.0815 |
|  | Indel857 | 0.1086 | 0.0263 |  | s2491 | 0.0042 | 0.0815 |
|  | Indel869 | 0.0212 | 0.0536 |  | s2492 | 0.0212 | 0.0536 |
|  | s1108 | 0.0212 | 0.0536 |  | s2634 | 0.0975 | 0.0281 |
|  | s1162 | 0.0212 | 0.0536 |  | s2673 | 0.0042 | 0.0815 |
|  | s1301 | 0.0212 | 0.0536 | *GmFT2a* | s125 | 0.5034 | 0.0039 |
|  | Indel1318 | 0.0212 | 0.0536 |  | s454 | 0.1291 | 0.0197 |
|  | s1326 | 0.0212 | 0.0536 |  | s4645 | 8.96E-04 | 0.0911 |
|  | s1351 | 0.0212 | 0.0536 | *GmFT2b* | s755 | 2.31E-15 | 0.4193 |
|  | Indel1364 | 0.0212 | 0.0536 |  | s1961 | 2.31E-15 | 0.4193 |
|  | s1373 | 0.0212 | 0.0536 |  | s2904 | 3.07E-05 | 0.1397 |
|  | s1378 | 0.0212 | 0.0536 | *GmFT5a* | s1129 | 3.37E-07 | 0.1886 |
|  | s1379 | 0.0212 | 0.0536 |  | s1487 | 0.0341 | 0.0354 |
|  | s1415 | 0.1086 | 0.0263 |  | Indel1914 | 0.0056 | 0.0597 |
|  | s1433 | 0.0212 | 0.0536 | *GmFT5b* | s62 | 1.28E-06 | 0.1994 |
|  | s1536 | 0.0212 | 0.0536 |  | s80 | 1.28E-06 | 0.1994 |
|  | s1551 | 0.0212 | 0.0536 |  | s160 | 1.28E-06 | 0.1994 |
|  | s1553 | 0.1086 | 0.0263 |  | s346 | 1.28E-06 | 0.1994 |
|  | s1582 | 0.1354 | 0.0228 |  | s539 | 1.28E-06 | 0.1994 |
|  | Indel1593 | 0.1354 | 0.0228 |  | s553 | 1.28E-06 | 0.1994 |
|  | Indel1636 | 0.0212 | 0.0536 |  | s554 | 1.28E-06 | 0.1994 |
|  | Indel1674 | 0.0056 | 0.1026 |  | s587 | 1.28E-06 | 0.1994 |
|  | s1683 | 0.0212 | 0.0536 |  | s707 | 1.28E-06 | 0.1994 |
|  | s1731 | 0.1086 | 0.0263 |  | s1032 | 1.28E-06 | 0.1994 |
|  | s1787 | 0.1086 | 0.0263 |  | s1163 | 1.28E-06 | 0.1994 |
|  | s1867 | 0.0042 | 0.0815 |  | s1344 | 1.28E-06 | 0.1994 |
|  | s1888 | 0.0042 | 0.0815 |  | s1558 | 1.28E-06 | 0.1994 |
|  | s1889 | 0.0042 | 0.0815 |  | s1662 | 1.28E-06 | 0.1994 |
|  | Indel1914 | 0.0212 | 0.0536 |  | s1856 | 1.28E-06 | 0.1994 |
|  | s2075 | 0.0042 | 0.0815 |  | s2194 | 1.28E-06 | 0.1994 |
|  | s2077 | 0.0212 | 0.0536 |  | s2418 | 1.28E-06 | 0.1994 |
|  | s2094 | 0.0212 | 0.0536 |  | s2435 | 1.28E-06 | 0.1994 |

Note: Ve-R1 represents the days from emergence to the beginning of bloom.
