# Supplementary material for: Natural variations of FT family genes in soybean varieties covering a wide range of maturity groups
Source: BMC Genomics. 2019 Mar 20;20:230. doi: 10.1186/s12864-019-5577-5 (PMC6425728; doi:10.1186/s12864-019-5577-5)
Supplement: Supplementary file 5 — Table S5. Polymorphic sites of the 10 soybean FT family genes from the present study. (DOCX 56 kb) [file 12864_2019_5577_MOESM5_ESM.docx]

**Table S5. Polymorphic sites of the 10 soybean *FT* family genes from the present study**

| **Gene** | **Chromosome** | **Polymorphic sites** | **Position in genome** | **Comparision to  Phytozome v11** | **Ref** | **Variant** | **Effect** |
| --- | --- | --- | --- | --- | --- | --- | --- |
|  |  |  |  |  |  |  |  |
| *GmFT1a* (*Glyma.18G298900*) | Chr18 | s337 | 57653720 | New | G | A | 5'UTR |
|  |  |  |  |  |  |  |  |
|  | Chr18 | s386 | 57653769 | New | T | G | Exon1 |
|  | Chr18 | s779 | 57654162 | New | G | A | Intron2 |
|  | Chr18 | s877 | 57654260 | New | C | G | Intron2 |
|  | Chr18 | s878 | 57654261 | New | G | A | Intron2 |
|  | Chr18 | Indel880 | 57654262 | New | T | TC_72bp_ | Intron2 |
|  | Chr18 | s954 | 57654265 | New | G | A | Intron2 |
|  | Chr18 | s955 | 57654266 | Common | T | G, A | Intron2 |
|  | Chr18 | s966 | 57654277 | New | G | A | Intron2 |
|  | Chr18 | s973 | 57654284 | New | C | T | Intron2 |
|  | Chr18 | s978 | 57654289 | New | C | T | Intron2 |
|  | Chr18 | s981 | 57654292 | New | C | T | Intron2 |
|  | Chr18 | s983 | 57654294 | New | A | G | Intron2 |
|  | Chr18 | s994 | 57654305 | New | T | A | Intron2 |
|  | Chr18 | s1000 | 57654311 | Common | T | C | Intron2 |
|  | Chr18 | s1001 | 57654312 | New | A | G | Intron2 |
|  | Chr18 | s1018 | 57654329 | New | G | A | Intron2 |
|  | Chr18 | s1022 | 57654333 | Common | T | C | Intron2 |
|  | Chr18 | s1035 | 57654346 | Common | G | A | Intron2 |
|  | Chr18 | s1040 | 57654351 | New | T | C | Intron2 |
|  | Chr18 | s1042 | 57654353 | New | C | T | Intron2 |
|  | Chr18 | Indel1046 | 57654355 | New | TC_31bp_ | T, TA_19bp,_ AC_31bp_ | Intron2 |
|  | Chr18 | Indel1059 | 57654368 | New | TT_12bp_ | T | Intron2 |
|  | Chr18 | s1077 | 57654388 | New | T | C | Intron2 |
|  | Chr18 | s1085 | 57654396 | New | A | T | Intron2 |
|  | Chr18 | s1088 | 57654399 | New | C | T | Intron2 |
|  | Chr18 | s1095 | 57654406 | New | A | G | Intron2 |
|  | Chr18 | s1098 | 57654409 | Common | A | G | Intron2 |
|  | Chr18 | s1108 | 57654419 | Common | C | T | Intron2 |
|  | Chr18 | s1120 | 57654431 | New | C | T | Intron2 |
|  | Chr18 | s1131 | 57654443 | Common | C | T | Intron2 |
|  | Chr18 | s1149 | 57654461 | Common | C | T | Intron2 |
|  | Chr18 | s1154 | 57654466 | New | A | G | Intron2 |
|  | Chr18 | s1218 | 57654530 | Common | A | G | Intron2 |
|  | Chr18 | s1228 | 57654540 | New | C | T | Intron2 |
|  | Chr18 | s1259 | 57654572 | Common | C | T | Intron2 |
|  | Chr18 | s1265 | 57654578 | New | A | G | Intron2 |
|  | Chr18 | s1284 | 57654597 | Common | G | A | Intron2 |
|  | Chr18 | s1285 | 57654598 | New | G | T, A | Intron2 |
|  | Chr18 | s1291 | 57654604 | New | T | C | Intron2 |
|  | Chr18 | s1294 | 57654607 | Common | G | A | Intron2 |
|  | Chr18 | s1310 | 57654623 | New | A | T, G | Intron2 |
|  | Chr18 | Indel1315 | 57654628 | New | TTTTT | TATTATTT, TATTATTTTA, T | Intron2 |
|  | Chr18 | s1325 | 57654633 | New | A | T | Intron2 |
|  | Chr18 | s1339 | 57654647 | New | G | A | Intron2 |
|  | Chr18 | s1350 | 57654658 | New | T | C | Intron2 |
|  | Chr18 | s1357 | 57654665 | New | A | G | Intron2 |
|  | Chr18 | s1410 | 57654718 | New | T | C | Intron2 |
|  | Chr18 | s1419 | 57654727 | New | G | A | Intron2 |
|  | Chr18 | s1424 | 57654732 | New | C | T | Intron2 |
|  | Chr18 | s1432 | 57654740 | New | A | G | Intron2 |
|  | Chr18 | s1444 | 57654752 | New | T | C | Intron2 |
|  | Chr18 | Indel1472 | 57654779 | New | G | GG | Intron2 |
|  | Chr18 | s1475 | 57654782 | New | T | G | Intron2 |
|  | Chr18 | s1494 | 57654801 | New | T | A | Intron2 |
|  | Chr18 | Indel1567 | 57654874 | New | TAATCT | T | Intron2 |
|  | Chr18 | s1789 | 57655096 | Common | T | C | Intron2 |
|  | Chr18 | Indel1887 | 57655193 | New | T | TT | Intron2 |
|  | Chr18 | Indel1930 | 57655235 | New | A | AA | Intron2 |
|  | Chr18 | s2100 | 57655405 | New | G | A | Intron2 |
|  | Chr18 | s2110 | 57655415 | Common | T | C | Intron2 |
|  | Chr18 | s2404 | 57655709 | Common | C | G | Intron2 |
|  | Chr18 | s2556 | 57655861 | Common | A | T | Intron2 |
|  | Chr18 | s2609 | 57655914 | Common | C | T | Intron2 |
|  | Chr18 | s2669 | 57655974 | New | A | C | Intron2 |
|  | Chr18 | s2694 | 57655999 | New | G | A | Intron2 |
|  | Chr18 | s2731 | 57656036 | New | C | T | Intron2 |
|  | Chr18 | Indel2754 | 57656058 | New | T | TAAAT | Intron2 |
|  | Chr18 | Indel2758 | 57656059 | New | TA | T | Intron2 |
|  | Chr18 | s2769 | 57656070 | New | A | T | Intron2 |
|  | Chr18 | s2774 | 57656075 | New | A | G | Intron2 |
|  | Chr18 | s2786 | 57656087 | New | T | A | Intron2 |
|  | Chr18 | s2865 | 57656166 | New | C | T | Intron2 |
|  | Chr18 | Indel2870 | 57656170 | New | T | TT | Intron2 |
|  | Chr18 | Indel2878 | 57656178 | New | AA | A | Intron2 |
|  | Chr18 | s2879 | 57656179 | New | T | A | Intron2 |
|  | Chr18 | s2884 | 57656184 | New | G | A | Intron2 |
|  | Chr18 | s2892 | 57656192 | New | G | T | Intron2 |
|  | Chr18 | s2924 | 57656224 | New | T | A | Intron2 |
|  | Chr18 | s3107 | 57656407 | New | T | A | Intron2 |
|  | Chr18 | s3111 | 57656411 | New | T | A | Intron2 |
|  | Chr18 | s3112 | 57656412 | New | A | C | Intron2 |
|  | Chr18 | s3120 | 57656420 | New | G | A | Intron2 |
|  | Chr18 | s3147 | 57656447 | New | T | C | Intron2 |
|  | Chr18 | s3168 | 57656468 | New | C | T | Intron2 |
|  | Chr18 | s3200 | 57656500 | Common | A | G | Intron2 |
|  | Chr18 | s3218 | 57656518 | New | C | T | Intron2 |
|  | Chr18 | s3246 | 57656546 | New | C | T | Intron2 |
|  | Chr18 | s3249 | 57656549 | New | C | T | Intron2 |
|  | Chr18 | s3266 | 57656566 | Common | C | T | Intron2 |
|  | Chr18 | s3344 | 57656644 | Common | A | G | Intron2 |
|  | Chr18 | Indel3384 | 57656684 | New | CCAC | C | Intron2 |
|  | Chr18 | s3389 | 57656689 | Common | G | C | Intron2 |
|  | Chr18 | s3477 | 57656777 | Common | T | C | Intron2 |
|  | Chr18 | Indel3478 | 57656778 | New | TAA | C, CAA | Intron2 |
|  | Chr18 | s3486 | 57656786 | Common | C | T | Intron2 |
|  | Chr18 | s3574 | 57656874 | Common | G | T | Intron2 |
|  | Chr18 | s3755 | 57657055 | Common | A | G | Intron2 |
|  | Chr18 | Indel3756 | 57657056 | New | ATTTGCTC | A, GTTTGCTC | Intron2 |
|  | Chr18 | s4038 | 57657338 | New | A | G | Intron3 |
|  | Chr18 | s4136 | 57657436 | New | A | G | Intron3 |
|  | Chr18 | Indel4420 | 57657720 | New | 7A | 6A, 8A | Intron3 |
|  | Chr18 | Indels4455 | 57657754 | New | AA | A | Intron3 |
|  | Chr18 | s4501 | 57657800 | New | C | A | Intron3 |
|  | Chr18 | Indel4654 | 57657953 | New | AA | A | Intron3 |
|  | Chr18 | s4757 | 57658056 | Common | T | A | Intron3 |
|  | Chr18 | s4851 | 57658150 | New | C | A | Intron3 |
|  | Chr18 | s4912 | 57658211 | New | G | C | Intron3 |
|  | Chr18 | s4920 | 57658219 | New | C | A | Intron3 |
|  | Chr18 | s5000 | 57658299 | New | T | A | Intron3 |
|  | Chr18 | Indel5188 | 57658486 | New | C | CTGGTAACATC | Intron3 |
|  | Chr18 | s5227 | 57658516 | Common | T | G | Exon4 |
|  | Chr18 | Indel5261 | 57658550 | New | GA | G | Exon4 |
|  | Chr18 | Indel5278 | 57658567 | New | TT | T | Exon4 |
|  | Chr18 | s5298 | 57658587 | Common | G | C | Exon4 |
|  | Chr18 | s5309 | 57658598 | New | C | T | Exon4 |
|  | Chr18 | s5322 | 57658611 | Common | G | C | Exon4 |
|  | Chr18 | s5323 | 57658612 | Common | C | G | Exon4 |
|  | Chr18 | s5324 | 57658613 | New | T | C | Exon4 |
|  | Chr18 | s5325 | 57658614 | New | G | T | Exon4 |
|  | Chr18 | Indel5337 | 57658626 | New | TT | T | Exon4 |
|  | Chr18 | s5344 | 57658633 | Common | T | C | Exon4 |
|  | Chr18 | Indel5353 | 57658642 | Common | TT | T | Exon4 |
|  | Chr18 | s5373 | 57658662 | Common | G | A | Exon4 |
|  | Chr18 | s5440 | 57658729 | New | A | G | Exon4 |
|  | Chr18 | s5708 | 57658997 | Common | A | T | 3'UTR |
|  | Chr18 | s5893 | 57659182 | New | T | C | 3'UTR |
|  | Chr18 | s5926 | 57659215 | Common | T | A | 3'UTR |
|  | Chr18 | s125 | 57670585 | Common | T | A | 5'UTR |
| *GmFT1b* (*Glyma.18G299000*) | Chr18 | s126 | 57670586 | Common | G | A | 5'UTR |
|  |  |  |  |  |  |  |  |
|  | Chr18 | s220 | 57670680 | Common | T | A | Exon1 |
|  | Chr18 | s387 | 57670847 | Common | C | T | intron1 |
|  | Chr18 | s421 | 57670881 | Common | A | C | intron1 |
|  | Chr18 | s627 | 57671087 | Common | A | C | intron2 |
|  | Chr18 | s644 | 57671104 | Common | T | C | intron2 |
|  | Chr18 | s670 | 57671130 | Common | T | A | intron2 |
|  | Chr18 | Indel719 | 57671179 | New | 9T | 8T, 10T | intron2 |
|  | Chr18 | Indel730 | 57671188 | New | T | TATT | intron2 |
|  | Chr18 | s736 | 57671192 | Common | T | C | intron2 |
|  | Chr18 | s775 | 57671231 | New | A | G | intron2 |
|  | Chr18 | Indel790 | 57671245 | New | T | TTGTCTTCTGT | intron2 |
|  | Chr18 | s805 | 57671251 | New | T | C | intron2 |
|  | Chr18 | Indel857 | 57671303 | New | TT | T | intron2 |
|  | Chr18 | Indel869 | 57671315 | New | TCTTTT | T | intron2 |
|  | Chr18 | Indel916 | 57671362 | New | GG | G | intron2 |
|  | Chr18 | s943 | 57671389 | Common | G | T | intron2 |
|  | Chr18 | s990 | 57671436 | Common | A | T | intron2 |
|  | Chr18 | s1083 | 57671529 | Common | T | C | intron2 |
|  | Chr18 | s1108 | 57671554 | New | A | C | intron2 |
|  | Chr18 | s1162 | 57671608 | New | A | T | intron2 |
|  | Chr18 | s1198 | 57671644 | New | A | T | intron2 |
|  | Chr18 | s1243 | 57671689 | Common | C | T | intron2 |
|  | Chr18 | s1254 | 57671700 | New | C | T | intron2 |
|  | Chr18 | s1255 | 57671701 | New | A | G | intron2 |
|  | Chr18 | s1301 | 57671747 | Common | T | C | intron2 |
|  | Chr18 | Indel1318 | 57671764 | New | AA | A | intron2 |
|  | Chr18 | s1326 | 57671772 | New | T | C | intron2 |
|  | Chr18 | s1351 | 57671797 | New | T | G | intron2 |
|  | Chr18 | Indel1364 | 57671809 | New | T | TACTTTCTTA | intron2 |
|  | Chr18 | s1373 | 57671810 | New | C | T | intron2 |
|  | Chr18 | s1378 | 57671815 | New | A | G | intron2 |
|  | Chr18 | s1379 | 57671816 | New | T | G | intron2 |
|  | Chr18 | s1415 | 57671852 | New | A | G | intron2 |
|  | Chr18 | s1423 | 57671860 | Common | T | C | intron2 |
|  | Chr18 | s1433 | 57671870 | New | A | G | intron2 |
|  | Chr18 | s1473 | 57671910 | Common | C | A | intron2 |
|  | Chr18 | s1536 | 57671973 | New | G | A | intron2 |
|  | Chr18 | s1551 | 57671988 | New | G | A | intron2 |
|  | Chr18 | s1553 | 57671990 | New | A | C | intron2 |
|  | Chr18 | s1582 | 57672019 | New | A | C | intron2 |
|  | Chr18 | Indel1593 | 57672030 | New | CC_31bp_ | C | intron2 |
|  | Chr18 | Indel1625 | 57672062 | New | GT_30bp_ | G | intron2 |
|  | Chr18 | Indel1629 | 57672066 | New | TTCA | T | intron2 |
|  | Chr18 | Indel1636 | 57672072 | New | A | ACAAA | intron2 |
|  | Chr18 | Indel1674 | 57672107 | Common | CT | C, CC | intron2 |
|  | Chr18 | s1683 | 57672116 | New | A | G | intron2 |
|  | Chr18 | s1684 | 57672117 | New | C | T | intron2 |
|  | Chr18 | s1692 | 57672125 | New | C | T | intron2 |
|  | Chr18 | s1707 | 57672140 | Common | A | T | intron2 |
|  | Chr18 | Indel1720 | 57672153 | New | A | CA, AAA, AA | intron2 |
|  | Chr18 | s1731 | 57672162 | New | T | A | intron2 |
|  | Chr18 | s1754 | 57672185 | New | A | C | intron2 |
|  | Chr18 | s1867 | 57672197 | New | C | C | intron2 |
|  | Chr18 | s1787 | 57672218 | Common | A | C | intron2 |
|  | Chr18 | Indel1840 | 57672271 | New | 7A | 6A, 8A | intron2 |
|  | Chr18 | s1888 | 57672318 | New | T | A | intron2 |
|  | Chr18 | s1889 | 57672319 | New | A | T | intron2 |
|  | Chr18 | Indel1894 | 57672324 | New | TA | A | intron2 |
|  | Chr18 | Indel1896 | 57672325 | New | T | TAT_9AT_ | intron2 |
|  | Chr18 | Indel1914 | 57672325 | New | T | TT_138bp_ | intron2 |
|  | Chr18 | s2075 | 57672350 | Common | T | C | intron2 |
|  | Chr18 | s2077 | 57672352 | New | C | T | intron2 |
|  | Chr18 | s2094 | 57672369 | Common | T | C | intron2 |
|  | Chr18 | s2111 | 57672386 | Common | T | C | intron2 |
|  | Chr18 | s2121 | 57672396 | New | A | G | intron2 |
|  | Chr18 | Indel2130 | 57672404 | New | T | TAT | intron2 |
|  | Chr18 | s2139 | 57672412 | Common | A | G | intron2 |
|  | Chr18 | s2161 | 57672434 | Common | C | T | intron2 |
|  | Chr18 | s2168 | 57672441 | Common | G | T | intron2 |
|  | Chr18 | s2174 | 57672447 | Common | C | T | intron2 |
|  | Chr18 | s2178 | 57672451 | Common | G | C | intron2 |
|  | Chr18 | s2200 | 57672473 | Common | C | T | Exon3 |
|  | Chr18 | s2248 | 57672521 | Common | C | T | intron3 |
|  | Chr18 | Indel2252 | 57672525 | New | TT | T, TTTT | intron3 |
|  | Chr18 | s2264 | 57672535 | Common | T | C | intron3 |
|  | Chr18 | s2311 | 57672582 | Common | C | A | intron3 |
|  | Chr18 | s2364 | 57672635 | Common | T | C | intron3 |
|  | Chr18 | s2380 | 57672651 | Common | C | A | intron3 |
|  | Chr18 | s2388 | 57672659 | Common | C | T | intron3 |
|  | Chr18 | s2396 | 57672667 | Common | T | C | intron3 |
|  | Chr18 | Indel2409 | 57672679 | New | A | ATTTTATAC | intron3 |
|  | Chr18 | s2437 | 57672700 | Common | C | T | Exon4 |
|  | Chr18 | s2443 | 57672706 | Common | G | A | Exon4 |
|  | Chr18 | s2491 | 57672754 | Common | T | C | Exon4 |
|  | Chr18 | s2492 | 57672755 | Common | C | A | Exon4 |
|  | Chr18 | s2570 | 57672833 | Common | A | G | Exon4 |
|  | Chr18 | s2618 | 57672881 | Common | A | C | Exon4 |
|  | Chr18 | s2634 | 57672897 | Common | A | C | Exon4 |
|  | Chr18 | s2669 | 57672932 | New | A | C | 3'UTR |
|  | Chr18 | s2673 | 57672966 | New | A | G | 3'UTR |
|  | Chr18 | s2674 | 57672967 | New | T | C | 3'UTR |
|  | Chr16 | Indel12 | 31110010 | New | GAAAGCATAAG | G | 5'UTR |
|  | Chr16 | s125 | 31110123 | New | A | T | Exon1 |
| *GmFT2a* (*Glyma.16G150700*) | Chr16 | s276 | 31110274 | Common | C | T | Intron1 |
|  |  |  |  |  |  |  |  |
|  | Chr16 | s350 | 31110348 | Common | T | G | Intron1 |
|  | Chr16 | s371 | 31110369 | New | T | C | Intron1 |
|  | Chr16 | s454 | 31110452 | New | A | C | Intron1 |
|  | Chr16 | s459 | 31110457 | Common | A | T | Intron1 |
|  | Chr16 | s766 | 31110764 | Common | T | C | Intron1 |
|  | Chr16 | Indel997 | 31110994 | New | A | ATATA | Intron1 |
|  | Chr16 | s1013 | 31111017 | New | C | T | Intron1 |
|  | Chr16 | s1039 | 31111043 | New | T | A | Intron1 |
|  | Chr16 | s1048 | 31111050 | New | T | C | Intron1 |
|  | Chr16 | Indel1336 | 31111330 | New | AA | A | Intron3 |
|  | Chr16 | s1355 | 31111349 | New | A | G | Intron3 |
|  | Chr16 | Indel1472 | 31111466 | New | TA | T | Intron3 |
|  | Chr16 | s1521 | 31111515 | Common | T | C | Intron3 |
|  | Chr16 | Indel1539 | 31111533 | New | CATGC | C | Intron3 |
|  | Chr16 | s1621 | 31111615 | Common | C | G | Intron3 |
|  | Chr16 | s1794 | 31111788 | Common | G | A | Intron3 |
|  | Chr16 | Indel1848 | 31111841 | New | A | AA | Intron3 |
|  | Chr16 | s2069 | 31112062 | New | T | A | Intron3 |
|  | Chr16 | Indel2194 | 31112187 | New | TA | A | Intron3 |
|  | Chr16 | Indel2673 | 31112666 | New | 11T | 9T, 10T | Intron3 |
|  | Chr16 | s2677 | 31112670 | New | T | A | Intron3 |
|  | Chr16 | s2701 | 31112694 | New | A | C | Intron3 |
|  | Chr16 | Indel2874 | 31112865 | New | TTT | T | Intron3 |
|  | Chr16 | Indel3094 | 31113084 | New | A | AA, AAA | Intron3 |
|  | Chr16 | s3191 | 31113180 | New | A | G | Intron3 |
|  | Chr16 | s3253 | 31113242 | New | T | A | Intron3 |
|  | Chr16 | s3261 | 31113250 | Common | G | C | Intron3 |
|  | Chr16 | s3675 | 31113664 | Common | C | T | Intron3 |
|  | Chr16 | Indel3787 | 31113775 | New | T | TT | Intron3 |
|  | Chr16 | s3833 | 31113821 | Common | C | A | Intron3 |
|  | Chr16 | Indel3841 | 31113829 | New | AAAGAAAAAA | A | Intron3 |
|  | Chr16 | s3964 | 31113952 | New | A | C | Intron3 |
|  | Chr16 | s4074 | 31114062 | Common | C | T | Intron3 |
|  | Chr16 | s4082 | 31114070 | New | T | A | Intron3 |
|  | Chr16 | Indel4106 | 31114094 | New | TT | T | Intron3 |
|  | Chr16 | s4269 | 31114257 | New | T | C | Intron3 |
|  | Chr16 | s4276 | 31114264 | New | G | T | Intron3 |
|  | Chr16 | s4281 | 31114269 | New | T | G | Intron3 |
|  | Chr16 | s4307 | 31114295 | New | C | T | Intron3 |
|  | Chr16 | s4335 | 31114323 | New | T | C | Intron3 |
|  | Chr16 | s4352 | 31114340 | New | A | G | Intron3 |
|  | Chr16 | s4420 | 31114408 | Common | G | A | Intron3 |
|  | Chr16 | s4645 | 31114633 | New | G | A | Exon4 |
|  | Chr16 | s4671 | 31114659 | Common | G | A | 3'UTR |
|  | Chr16 | Indel4699 | 31114686 | New | A | AA | 3'UTR |
|  | Chr16 | s4908 | 31114895 | New | G | C | 3'UTR |
|  | Chr16 | s4943 | 31114930 | Common | G | T | 3'UTR |
|  | Chr16 | s5365 | 31115352 | Common | T | G | Downstream |
|  | Chr16 | s189 | 31149002 | New | T | C | Exon1 |
|  | Chr16 | s222 | 31149035 | New | A | T | Intron1 |
| *GmFT2b* (*Glyma.16G151000*) | Chr16 | s409 | 31149222 | New | G | A | Exon2 |
|  |  |  |  |  |  |  |  |
|  | Chr16 | s577 | 31149390 | New | C | T | Intron2 |
|  | Chr16 | Indel667 | 31149479 | New | A | AA | Intron3 |
|  | Chr16 | Indel680 | 31149491 | New | T | TT | Intron3 |
|  | Chr16 | Indel705 | 31149515 | New | G | GG | Intron3 |
|  | Chr16 | s755 | 31149565 | Common | A | C | Intron3 |
|  | Chr16 | s762 | 31149572 | Common | T | A | Intron3 |
|  | Chr16 | Indel807 | 31149616 | New | G | GG | Intron3 |
|  | Chr16 | s867 | 31149676 | New | A | G | Intron3 |
|  | Chr16 | Indel883 | 31149691 | New | A | ATA | Intron3 |
|  | Chr16 | Indel893 | 31149699 | New | T | TT | Intron3 |
|  | Chr16 | Indel992 | 31149798 | New | AGTA | A | Intron3 |
|  | Chr16 | s1043 | 31149849 | New | T | G | Intron3 |
|  | Chr16 | s1099 | 31149905 | New | A | T | Intron3 |
|  | Chr16 | s1148 | 31149954 | New | A | G | Intron3 |
|  | Chr16 | s1180 | 31149986 | New | A | C | Intron3 |
|  | Chr16 | s1232 | 31150038 | Common | T | G | Intron3 |
|  | Chr16 | s1306 | 31150112 | New | G | A | Intron3 |
|  | Chr16 | s1307 | 31150113 | New | T | A | Intron3 |
|  | Chr16 | s1343 | 31150149 | New | G | C | Intron3 |
|  | Chr16 | s1384 | 31150190 | New | A | T | Intron3 |
|  | Chr16 | Indel1386 | 31150191 | New | A | ATATA | Intron3 |
|  | Chr16 | Indel1399 | 31150201 | New | AA | A | Intron3 |
|  | Chr16 | s1456 | 31150258 | Common | G | A | Intron3 |
|  | Chr16 | Indel1459 | 31150260 | New | G | GG | Intron3 |
|  | Chr16 | s1613 | 31150414 | New | C | T | Intron3 |
|  | Chr16 | s1629 | 31150430 | Common | C | T | Intron3 |
|  | Chr16 | s1654 | 31150455 | Common | A | C | Intron3 |
|  | Chr16 | Indel1692 | 31150493 | New | TATAT | T | Intron3 |
|  | Chr16 | s1723 | 31150524 | Common | A | T | Intron3 |
|  | Chr16 | Indel1731 | 31150532 | New | ATA | A | Intron3 |
|  | Chr16 | Indel1755 | 31150556 | New | AA | A, AAA | Intron3 |
|  | Chr16 | Indel1815 | 31150615 | New | AACA | A | Intron3 |
|  | Chr16 | s1948 | 31150748 | Common | A | T | Intron3 |
|  | Chr16 | Indel1956 | 31150755 | New | A | AAGA | Intron3 |
|  | Chr16 | s1961 | 31150758 | Common | C | T | Intron3 |
|  | Chr16 | s2073 | 31150870 | Common | T | A | Intron3 |
|  | Chr16 | Indel2143 | 31150939 | New | G | GTTG | Intron3 |
|  | Chr16 | s2213 | 31151007 | Common | T | C | Intron3 |
|  | Chr16 | s2319 | 31151113 | Common | A | T | Intron3 |
|  | Chr16 | s2579 | 31151372 | New | T | C | Intron3 |
|  | Chr16 | s2580 | 31151373 | New | A | G | Intron3 |
|  | Chr16 | s2587 | 31151380 | New | A | G | Intron3 |
|  | Chr16 | Indel2588 | 31151381 | New | AA | G | Intron3 |
|  | Chr16 | s2612 | 31151405 | New | C | T | Intron3 |
|  | Chr16 | s2633 | 31151426 | New | C | T | Intron3 |
|  | Chr16 | s2637 | 31151430 | New | A | C | Intron3 |
|  | Chr16 | Indel2547 | 31151440 | New | T | TT | Intron3 |
|  | Chr16 | Indel2648 | 31151440 | New | T | TT | Intron3 |
|  | Chr16 | s2650 | 31151442 | New | A | T | Intron3 |
|  | Chr16 | Indel2651 | 31151442 | New | A | TTTTTTCTT | Intron3 |
|  | Chr16 | s2702 | 31151486 | Common | T | C | Intron3 |
|  | Chr16 | s2711 | 31151495 | Common | T | C | Intron3 |
|  | Chr16 | s2826 | 31151610 | New | G | A | Intron3 |
|  | Chr16 | s2904 | 31151688 | Common | G | A | Exon4 |
|  | Chr16 | s2908 | 31151692 | Common | A | G | Exon4 |
|  | Chr16 | s2928 | 31151712 | New | A | G | Exon4 |
|  | Chr16 | s2983 | 31151767 | Common | T | C | Exon4 |
|  | Chr16 | s3040 | 31151824 | Common | T | C | Exon4 |
|  | Chr16 | s208 | 4164726 | New | T | C | Exon1 |
|  | Chr16 | s580 | 4164354 | Common | A | G | Intron2 |
| *GmFT3a* (*Glyma.16G044200*) | Chr16 | Indel712 | 4164222 | New | GTATA | G | Intron3 |
|  |  |  |  |  |  |  |  |
|  | Chr16 | s822 | 4164112 | Common | T | G | Intron3 |
|  | Chr16 | s1391 | 4163543 | Common | T | A | Intron3 |
|  | Chr16 | s1794 | 4163140 | New | G | T | Intron3 |
|  | Chr16 | s1953 | 4162981 | Common | G | A | Intron3 |
|  | Chr16 | s2129 | 4162805 | Common | G | A | Intron3 |
|  | Chr16 | s2259 | 4162675 | New | T | C | Intron3 |
|  | Chr16 | s2301 | 4162633 | New | A | G | Intron3 |
|  | Chr16 | s2398 | 4162536 | New | A | C | Intron3 |
|  | Chr19 | Indel521 | 36031151 | New | A | ATA | Intron3 |
|  | Chr19 | s547 | 36031176 | Common | T | G | Intron3 |
| *GmFT3b* (*Glyma.19G108100*) | Chr19 | s581 | 36031210 | New | T | A | Intron3 |
|  |  |  |  |  |  |  |  |
|  | Chr19 | s588 | 36031217 | New | A | T | Intron3 |
|  | Chr19 | s596 | 36031225 | New | G | A | Intron3 |
|  | Chr19 | Indel839 | 36031268 | New | ATATA | A | Intron3 |
|  | Chr19 | s640 | 36031269 | New | A | G | Intron3 |
|  | Chr19 | s866 | 36031495 | New | T | C | Intron3 |
|  | Chr19 | s876 | 36031505 | New | T | C | Intron3 |
|  | Chr19 | s908 | 36031537 | New | C | G | Intron3 |
|  | Chr19 | s1167 | 36031796 | New | T | C | Intron3 |
|  | Chr19 | Indel1174 | 36031802 | New | 6T | 7T, 8T | Intron3 |
|  | Chr19 | s1349 | 36031976 | New | C | T | Intron3 |
|  | Chr19 | s1453 | 36032080 | Common | A | G | Intron3 |
|  | Chr19 | Indel1494 | 36032120 | New | T | TT | Intron3 |
|  | Chr19 | s1764 | 36032390 | Common | T | A | Intron3 |
|  | Chr19 | s1774 | 36032400 | Common | C | G | Intron3 |
|  | Chr19 | s1786 | 36032412 | New | T | C | Intron3 |
|  | Chr19 | s1860 | 36032486 | New | C | T | Intron3 |
|  | Chr19 | s1874 | 36032500 | New | A | T | Intron3 |
|  | Chr19 | s1879 | 36032505 | New | C | A | Intron3 |
|  | Chr19 | s1963 | 36032589 | New | A | T | Intron3 |
|  | Chr19 | s2033 | 36032659 | New | G | A | Exon4 |
|  | Chr19 | s2078 | 36032704 | New | T | G | Exon4 |
|  | Chr08 | s107 | 47459797 | New | C | G | 5'UTR |
| *GmFT4* (Glyma.08G363100) | Chr08 | Indel560 | 47459347 | New | 9TA | 6TA, 10TA | Intron2 |
|  |  |  |  |  |  |  |  |
|  | Chr08 | s580 | 47459328 | New | T | C | Intron2 |
|  | Chr08 | s934 | 47458974 | New | G | A | Intron2 |
|  | Chr08 | Indel1278 | 47458631 | New | T | TTA | Intron3 |
|  | Chr08 | Indel1351 | 47458559 | New | TT_19bp_ | T | Intron3 |
|  | Chr08 | Indel1651 | 47458260 | New | C | CA | 3'UTR |
|  | Chr08 | Indel1678 | 47458239 | New | T | TG | 3'UTR |
| *GmFT5a* (Glyma.16G044100) | Chr16 | Indel68 | 4137794 | New | A | AAT | Upstream |
|  |  |  |  |  |  |  |  |
|  | Chr16 | s441 | 4137422 | New | G | T | Intron1 |
|  | Chr16 | s921 | 4136942 | New | A | T | Intron2 |
|  | Chr16 | s1129 | 4136734 | Common | C | T | Intron3 |
|  | Chr16 | s1160 | 4136703 | New | C | T | Intron3 |
|  | Chr16 | Indel1185 | 4136679 | New | A | ATA | Intron3 |
|  | Chr16 | s1187 | 4136678 | New | G | T | Intron3 |
|  | Chr16 | Indel1213 | 4136652 | New | ATTGAT | A | Intron3 |
|  | Chr16 | s1305 | 4136560 | New | G | A | Exon4 |
|  | Chr16 | s1487 | 4136378 | Common | G | T | 3'UTR |
|  | Chr16 | s1531 | 4136334 | New | G | A | 3'UTR |
|  | Chr16 | Indel1577 | 4136288 | New | TA_15bp_ | T | 3'UTR |
|  | Chr16 | s1592 | 4136273 | New | A | G | 3'UTR |
|  | Chr16 | Indel1914 | 4135951 | New | AT_49bp_ | A | 3'UTR |
|  | Chr19 | s62 | 36049116 | New | T | C | 5'UTR |
|  | Chr19 | s80 | 36049134 | Common | G | T | 5'UTR |
| *GmFT5b* (Glyma.19G108200) | Chr19 | s160 | 36049214 | Common | A | G | Exon1 |
|  |  |  |  |  |  |  |  |
|  | Chr19 | Indel346 | 36049400 | New | GTAG | G | Intron1 |
|  | Chr19 | s539 | 36049593 | Common | T | G | Intron2 |
|  | Chr19 | s553 | 36049607 | Common | C | A | Intron2 |
|  | Chr19 | s554 | 36049608 | Common | G | C | Intron2 |
|  | Chr19 | s569 | 36049623 | Common | T | C | Intron2 |
|  | Chr19 | Indel587 | 36049640 | New | C | CC | Intron2 |
|  | Chr19 | s707 | 36049760 | Common | G | T | Intron2 |
|  | Chr19 | s1032 | 36050085 | Common | G | T | Intton3 |
|  | Chr19 | s1117 | 36050170 | Common | G | A | Intton3 |
|  | Chr19 | s1163 | 36050216 | Common | T | C | Intton3 |
|  | Chr19 | s1344 | 36050397 | Common | A | G | Intton3 |
|  | Chr19 | Indel1403 | 36050455 | New | A | AA | Intton3 |
|  | Chr19 | Indel1536 | 36050587 | New | T | TT | Intton3 |
|  | Chr19 | s1558 | 36050609 | Common | G | A | Intton3 |
|  | Chr19 | s1662 | 36050713 | Common | G | A | Intton3 |
|  | Chr19 | s1821 | 36050872 | Common | T | C | Intton3 |
|  | Chr19 | s1856 | 36050907 | Common | G | A | Intton3 |
|  | Chr19 | Indel1870 | 36050921 | New | TT | T | Intton3 |
|  | Chr19 | Indel1877 | 36050927 | New | T | TT_174bp_ | Intton3 |
|  | Chr19 | s2110 | 36050987 | Common | G | A | Intton3 |
|  | Chr19 | Indel2142 | 36051019 | New | TT | T | Intton3 |
|  | Chr19 | s2194 | 36051071 | Common | T | A | Intton3 |
|  | Chr19 | s2387 | 36051264 | Common | C | G | Intton3 |
|  | Chr19 | s2418 | 36051295 | Common | T | A | Intton3 |
|  | Chr19 | s2435 | 36051312 | Common | G | A | Intton3 |
|  | Chr08 | s74 | 47473451 | New | A | G | Upstream |
|  | Chr08 | s316 | 47473204 | New | A | G | Exon1 |
| *GmFT6* (Glyma08g47820) | Chr08 | s429 | 47473091 | Common | T | G | Intron1 |
|  |  |  |  |  |  |  |  |
|  | Chr08 | s446 | 47473074 | Common | T | G | Intron1 |
|  | Chr08 | s473 | 47473047 | New | G | A | Intron1 |
|  | Chr08 | s522 | 47472998 | New | T | A | Intron1 |
|  | Chr08 | Indel524 | 47472997 | New | C | CAT | Intron1 |
|  | Chr08 | s754 | 47472768 | Common | G | C | Intron2 |
|  | Chr08 | s1144 | 47472378 | Common | T | A | Intron2 |
|  | Chr08 | s1450 | 47472072 | Common | G | C | Intron2 |
|  | Chr08 | s1483 | 47472039 | New | A | C | Intron2 |
|  | Chr08 | s1487 | 47472035 | New | A | C | Intron2 |
|  | Chr08 | Indel1489 | 47472033 | New | CA | C | Intron2 |
|  | Chr08 | s1579 | 47471943 | New | T | A | Intron2 |
|  | Chr08 | s1582 | 47471940 | Common | C | T | Intron2 |
|  | Chr08 | Indel2064 | 47471459 | Common | C | CA_58bp_ | Intron2 |
|  | Chr08 | s2125 | 47471455 | New | T | G | Intron2 |
|  | Chr08 | Indel2244 | 47471337 | New | 12T | 11T, 13T | Intron2 |
|  | Chr08 | Indel3047 | 47470534 | New | TA | T | Intron2 |
|  | Chr08 | Indel3188 | 47470394 | New | G | GA | Intron2 |
|  | Chr08 | Indel3228 | 47470354 | New | CA | C | Intron2 |
|  | Chr08 | s4057 | 47469525 | Common | A | G | Intron2 |
|  | Chr08 | s4176 | 47469406 | New | G | T | Intron2 |
|  | Chr08 | s4231 | 47469351 | Common | A | T | Intron2 |
|  | Chr08 | s4641 | 47468941 | Common | G | A | Intron2 |
|  | Chr08 | s4826 | 47468756 | Common | G | A | Intron2 |
|  | Chr08 | s4924 | 47468658 | New | A | G, T | Intron2 |
|  | Chr08 | Indel4937 | 47468645 | New | AT | A | Intron2 |
|  | Chr08 | Indel4954 | 47468628 | New | TA | T | Intron2 |
|  | Chr08 | s5016 | 47468566 | New | A | G | Intron2 |
|  | Chr08 | s5305 | 47468277 | New | T | C | Intron2 |
|  | Chr08 | s5406 | 47468176 | Common | T | A | Intron2 |
|  | Chr08 | s5641 | 47467941 | New | T | G | Intron2 |
|  | Chr08 | s5655 | 47467927 | New | T | G | Intron2 |
|  | Chr08 | Indel5662 | 47467921 | New | G | GTTT | Intron2 |
|  | Chr08 | s5670 | 47467915 | New | T | G | Intron2 |
|  | Chr08 | s5983 | 47467602 | New | A | T | Intron3 |
|  | Chr08 | s6325 | 47467260 | New | C | G | Exon4 |

Note: “Common” means this variation was detected in both Phytozome v11 and the present study; “New” represents this variation was just detected in the present study.
