# Supplementary material for: Natural variations of FT family genes in soybean varieties covering a wide range of maturity groups
Source: BMC Genomics. 2019 Mar 20;20:230. doi: 10.1186/s12864-019-5577-5 (PMC6425728; doi:10.1186/s12864-019-5577-5)
Supplement: Supplementary file 6 — Table S6. Flowering time (Ve-R1) and growth duration (Ve-R7 and Ve-R8) data in Beijing, China, in 2015. (DOCX 21 kb) [file 12864_2019_5577_MOESM6_ESM.docx]

**Table S6. Flowering time (Ve-R1) and growth duration (Ve-R7 and Ve-R8) data in Beijing, China, in 2015**

| **Variety** | **Ve-R1** | **Ve-R7** | **Ve-R8** | **Variety** | | **Ve-R1** | **Ve-R7** | **Ve-R8** |
| --- | --- | --- | --- | --- | --- | --- | --- | --- |
| Star4/75 | 22.3±0.6 | 61.4±1.7 | 66.2±1.4 | | Williams 82 | 37.9±0.1 | 116.3±0.8 | 124.6±0.5 |
| R-4 | 27.4±2.6 | 66.9±5.4 | 74.4±7.9 | | Xudou 9 | 41.2±1.3 | 111.8±4.5 | 125.5±6.1 |
| Hujiao07-2479 | 22.2±0.2 | 67.2±1.0 | 72.2±1.2 | | Zhongdou 39 | 45.6±0.7 | 108.5±2.0 | 121.0±1.9 |
| Paula | 22.4±0.7 | 63.5±1.2 | 68.6±1.1 | | Zhonghuang13 | 45.8±0.4 | 111.8±1.1 | 117.3±2.4 |
| Linbei 8 | 22.5±1.3 | 64.8±2.6 | 69.0±2.1 | | Huaidou 9 | 46.6±0.5 | 120.1±8.0 | 129.6±3.0 |
| Maple Presto | 23.4±0.6 | 63.8±2.0 | 69.3±2.6 | | IL2 | 49.3±4.6 | 115.1±0.8 | 127.3±1.1 |
| Hujiao07-2123 | 23.7±0.5 | 69.1±3.9 | 76.6±10.3 | | Huachun 6 | 51.4±0.1 | 112.1±1.4 | 117.2±0.6 |
| R2 | 23.7±1.0 | 61.7±5.5 | 67.4±4.8 | | Flyer | 34.8±3.5 | 115.1±0.2 | 125.6±1.2 |
| Rassvet | 23.9±1.4 | 69.5±12.0 | 74.2±11.6 | | Omaha | 37.5±0.8 | 116.6±0.9 | 126.8±0.3 |
| Heihe 35 | 24.8±0.2 | 66.2±0.7 | 70.5±0.4 | | CF461 | 39.9±1.1 | 124.8±0.7 | 131.8±2.1 |
| OAC Vision | 24.8±2.0 | 69.7±3.4 | 74.6±4.0 | | Zheng 92116 | 43.4±0.9 | 113.1±2.4 | 124.2±1.1 |
| Dongnong41-C | 24.9±0.8 | 62.8±2.3 | 68.2±4.3 | | Jindou 39 | 44.4±1.9 | 119.1±2.5 | 125.8±1.6 |
| Jug 30 | 25.6±0.7 | 71.1±2.8 | 75.2±3.2 | | Calhoun | 46.3±0.6 | 118.6±0.8 | 126.6±0.1 |
| Mageva | 26.2±0.2 | 70.4±3.0 | 79.8±5.5 | | Shanning 16 | 46.3±3.8 | 117.5±2.3 | 126.9±1.9 |
| Dongnong36 | 26.3±1.3 | 68.2±4.2 | 74.5±7.0 | | Guandou 2 | 51.8±4.2 | 126.0±1.1 | 132.3±0.6 |
| Dongnong41 | 26.5±0.9 | 72.0±5.6 | 81.1±5.5 | | UA 4805 | 59.0±4.8 | 130.1±2.4 | 137.5±1.4 |
| Beidou 16 | 23.1±0.9 | 65.2±3.1 | 71.3±3.9 | | *Houzimao | 73.5±1.3 | 135.0±0.0 | 140.0±0.0 |
| Mengdou 11 | 24.2±1.2 | 67.2±0.9 | 70.1±0.6 | | Nathan | 53.2±2.1 | 125.7±1.1 | 137.8±0.5 |
| Dongnong 44 | 24.3±0.1 | 77.3±4.6 | 86.1±5.6 | | Holladay | 57.6±1.7 | 128.4±2.2 | 141.0±3.8 |
| McCall | 24.3±0.2 | 72.9±1.0 | 78.0±1.3 | | Hutcheson | 58.9±1.6 | 135.0±1.2 | 142.5±1.1 |
| Mengdou 32 | 24.4±0.4 | 67.0±0.9 | 70.2±1.2 | | R01-3474F | 59.9±1.8 | 133.9±1.5 | 143.8±1.4 |
| Maple Ridge | 24.5±0.3 | 68.6±0.9 | 74.2±0.7 | | Dian 86-4 | 63.4±0.6 | 134.3±3.0 | 140.9±4.1 |
| Daksoy | 26.1±1.2 | 74.6±1.6 | 79.1±2.5 | | TN04-5321 | 66.1±3.0 | 138.6±1.3 | 149.0±1.8 |
| Agassiz | 26.2±0.3 | 78.1±2.6 | 86.1±5.5 | | Shangdou 14 | 67.7±1.3 | 133.1±4.4 | 140.3±0.4 |
| Canatto | 29.5±4.9 | 64.0 | 68.0 | | *Diandou 7 | 71.7±7.5 | 140.0±2.8 | 145.0±2.8 |
| Heihe 18 | 22.2±0.3 | 71.5±1.2 | 74.6±2.8 | | Desha | 66.4±3.6 | 136.8±0.8 | 145.9±1.0 |
| Heihe 43 | 23.1±0.1 | 75.6±3.5 | 79.7±2.2 | | *Boggs | 68.7±2.1 | 145.1±0.3 | 150.7±1.0 |
| Hefeng 25 | 23.1±0.7 | 72.6±2.7 | 76.9±4.7 | | *Suxiandou 19 | 71.7±2.1 | 147.7±0.6 | 154.0±0.9 |
| Heihe 27 | 23.2±0.3 | 77.9±1.5 | 84.8±0.9 | | *G01-PR16 | 72.9±0.6 | 146.7±0.6 | 154.3±0.6 |
| Dengke 1 | 23.3±0.5 | 72.8±3.2 | 76.6±4.4 | | *Zhongdou 38 | 73.5±2.1 | 135.0±0.0 | 140.8±0.4 |
| Beidou 37 | 23.3±0.6 | 76.7±2.9 | 82.8±5.2 | | *Musen | 75.1±0.1 | 143.7±3.2 | 151.1±5.1 |
| Traill | 23.7±0.8 | 77.7±2.0 | 85.7±3.2 | | *D95-6271 | 75.7±1.8 | 144.0±2.6 | 148.7±3.1 |
| Fengshou 12 | 23.7±0.8 | 76.6±2.2 | 80.8±1.8 | | *Wuhuasiyuehuang | 79.0±0.9 | 142.6±1.8 | 151.5±1.6 |
| Dongnong 4 | 23.9±0.1 | 80.5±0.9 | 86.9±0.2 | | *Nannong 493/1 | 86.2±1.5 | 149.3±3.6 | 154.8±2.5 |
| Norpro | 24.5±1.1 | 86.3±8.0 | 93.0±9.5 | | *Hengyangbayueqing | 93.2±2.3 | 143.8±3.0 | 152.3±1.5 |
| Jiangmodou 1 | 24.7±2.7 | 75.8±7.4 | 89.4±20.7 | | *Benning | 69.5±1.4 | 149.6±2.5 | 155.0±1.0 |
| Barnes | 25.4±1.2 | 85.1±5.8 | 91.1±5.3 | | *Santee | 71.4±1.6 | 146.4±2.7 | 151.5±3.4 |
| Dawson | 27.0±1.1 | 89.7±2.1 | 100.8±3.1 | | *Stonewall | 72.6±0.6 | 150.6±0.5 | 155.7±0.3 |
| Chico | 29.5±3.5 | 75.1±5.9 | 89.0±18.7 | | *Hagood | 77.0±0.9 | 149.8±0.2 | 155.3±0.8 |
| Heinong 16 | 23.7±0.6 | 82.4±2.7 | 87.5±3.7 | | *Nanxiadou 25 | 83.1±2.7 | 155.7±0.5 | 160.8±1.3 |
| Heinong 26 | 24.3±0.4 | 86.9±3.8 | 90.5±3.1 | | *Tongshanbaopihuang | 87.4±1.6 | 146.9±1.5 | 153.7±2.4 |
| Suinong 14 | 25.5±1.1 | 98.8±2.9 | 106.3±2.2 | | *Huangfengwo | 90.8±1.0 | 148.1±0.4 | 153.6±1.3 |
| Kato | 25.8±0.5 | 89.0±4.4 | 99.5±3.4 | | *Dowling | 68.4±2.4 | 149.0±1.2 | 154.9±0.5 |
| Taixingheidou | 27.9±0.8 | 75.1±0.8 | 81.7±2.2 | | *Motte | 79.4±1.0 | 151.7±2.1 | 156.1±0.2 |
| Haroson | 29.0±1.9 | 96.8±6.5 | 108.0±2.6 | | *Prichard | 83.6±1.7 | >148.1 | NA |
| Tiefeng 19 | 29.9±0.2 | 102.6±0.5 | 106.5±2.0 | | *Aijiaoqing | 85.2±3.3 | 154.0±0.0 | 157.0±2.6 |
| Parker | 30.3±2.0 | 103.8±6.0 | 108.3±6.0 | | *Nandou 12 | 91.0±0.0 | NA | NA |
| Granite | 33.5±0.6 | 106.3±1.3 | 112.5±0.6 | | *Lanxidaqingdou | 91.5±2.8 | >156.7 | NA |
| NE1900 | 34.3±0.3 | 105.8±0.8 | 111.8±1.7 | | *Shangraodaqingsi | 98.0±0.9 | >156.9 | NA |
| Jilin 20 | 26.6±1.6 | 101.5±0.7 | 106.0±0.0 | | *Nandou 17 | 98.8±4.2 | >156.7 | NA |
| Holt | 29.2±0.7 | 107.0±0.6 | 115.0±1.3 | | *Guixia 3 | 103.2±1.7 | >157.0 | NA |
| Century 84 | 31.1±0.3 | 107.4±2.2 | 115.6±2.7 | | *Pinguohuangdou | 103.7±1.9 | >156.5 | NA |
| Olympus | 31.3±0.7 | 104.9±1.0 | 114.8±1.4 | | *Qiudou 1 | 107.0±2.3 | >157.0 | NA |
| LN92-7369 | 33.4±0.6 | 110.4±2.4 | 118.1±1.1 | | *Zigongdongdou | 120.5±5.6 | >157.0 | NA |
| IL1 | 41.4±1.4 | 100.4±2.7 | 113.7±3.4 | | *'IAC-8 | 76.9±2.1 | >156.5 | NA |
| Yongchengzihuadou | 42.3±0.5 | 106.8±1.6 | 115.6±0.6 | | *'UFV-3 | 101.1±0.8 | >156.5 | NA |
| Xiangchundou 24 | 43.2±0.7 | 105.5±0.5 | 114.2±0.6 | | *'FT-15 | 104.3±1.1 | >156.3 | NA |
| Tiefeng 33 | 27.4±1.5 | 112.3±1.4 | 119.8±6.0 | | *'Alamo | 109.3±1.6 | >156.6 | NA |
| Tiefeng 31 | 29.7±0.2 | 115.8±1.2 | 122.1±2.0 | | *I.C. 192 | 115.9±0.9 | >156.3 | NA |
| Jindou 19 | 34.1±0.4 | 114.3±1.2 | 122.7±0.9 | | *Jupiter | 117.6±1.4 | >157.0 | NA |
| Athow | 34.3±0.5 | 112.8±0.4 | 120.8±0.4 | | *CIGRAS-06 | 78.0±1.3 | >156.3 | NA |
| Zhonghuang30 | 35.1±2.6 | 109.8±2.2 | 116.7±2.2 | | *CIGRAS-51 | 78.6±1.2 | >156.5 | NA |
| KS3494 | 35.9±1.0 | 114.8±0.3 | 122.2±2.9 | | *Jiangledaqingdou | 95.1±5.5 | >157.0 | NA |
| LN89-5699 | 36.3±1.1 | 113.0±0.8 | 120.5±0.9 | |  |  |  |  |

Note: * The varieties were moved into greenhouse after October 1 in 2015 and were treated with short day (12 h light/12 h dark). “>” means that these varieties didn’t mature till November 9 and we collected data on their final developmental stage at that time.
