# Supplementary material for: Natural variations of FT family genes in soybean varieties covering a wide range of maturity groups
Source: BMC Genomics. 2019 Mar 20;20:230. doi: 10.1186/s12864-019-5577-5 (PMC6425728; doi:10.1186/s12864-019-5577-5)
Supplement: Supplementary file 7 — Table S7. Haplotypes of the 10 soybean FT family genes in 127 varieties covering 14 maturity groups. (DOCX 38 kb) [file 12864_2019_5577_MOESM7_ESM.docx]

**Table S7. Haplotypes of the 10 soybean *FT* family genes in 127 varieties covering 14 maturity groups**

| **Variety** | **Maturity Group** | ***GmFT1a*** | ***GmFT1b*** | ***GmFT2a*** | ***GmFT2b*** | ***GmFT3a*** | ***GmFT3b*** | ***GmFT4*** | ***GmFT5a*** | ***GmFT5b*** | ***GmFT6*** |
| --- | --- | --- | --- | --- | --- | --- | --- | --- | --- | --- | --- |
| Star4/75 | 0000 | / | 1b-H5 | 2a-H4 | / | 3a-H1 | 3b-H2 | 4-H2 | 5a-H3 | 5b-H2 | 6-H4 |
| R-4 | 0000 | 1a-H6 | 1b-H3 | 2a-H10 | 2b-H1 | 3a-H2 | 3b-H2 | 4-H2 | 5a-H4 | / | 6-H4 |
| Hujiao07-2479 | 000 | 1a-H1 | 1b-H5 | 2a-H6 | 2b-H3 | 3a-H2 | 3b-H4 | 4-H3 | 5a-H4 | 5b-H1 | 6-H1 |
| Paula | 000 | 1a-H9 | / | 2a-H6 | / | / | 3b-H2 | 4-H2 | 5a-H6 | 5b-H2 | 6-H4 |
| Linbei 8 | 000 | 1a-H5 | 1b-H3 | 2a-H6 | 2b-H3 | 3a-H1 | 3b-H6 | 4-H2 | 5a-H3 | 5b-H4 | 6-H4 |
| Maple Presto | 000 | 1a-H1 | / | 2a-H6 | 2b-H3 | 3a-H1 | 3b-H2 | 4-H2 | 5a-H2 | 5b-H2 | 6-H4 |
| R2 | 000 | 1a-H11 | 1b-H5 | 2a-H4 | 2b-H8 | 3a-H1 | 3b-H2 | 4-H2 | 5a-H3 | 5b-H2 | 6-H4 |
| Hujiao07-2123 | 000 | 1a-H1 | / | / | 2b-H3 | 3a-H2 | 3b-H3 | 4-H3 | 5a-H4 | 5b-H1 | 6-H1 |
| Rassvet | 000 | 1a-H6 | 1b-H3 | 2a-H6 | 2b-H3 | 3a-H1 | 3b-H6 | 4-H2 | 5a-H3 | 5b-H5 | 6-H4 |
| OAC Vision | 000 | 1a-H6 | 1b-H3 | / | / | 3a-H1 | 3b-H4 | 4-H2 | 5a-H3 | 5b-H1 | 6-H4 |
| Heihe 35 | 000 | 1a-H10 | / | 2a-H6 | 2b-H3 | 3a-H2 | 3b-H7 | 4-H1 | 5a-H4 | 5b-H4 | 6-H2 |
| Dongnong41-C | 000 | 1a-H5 | 1b-H3 | / | 2b-H1 | 3a-H2 | 3b-H7 | 4-H2 | 5a-H4 | 5b-H4 | 6-H4 |
| Jug 30 | 000 | 1a-H11 | / | 2a-H6 | 2b-H3 | / | 3b-H2 | 4-H2 | 5a-H3 | 5b-H2 | 6-H4 |
| Mageva | 000 | / | 1b-H3 | 2a-H6 | 2b-H3 | 3a-H1 | 3b-H7 | 4-H2 | 5a-H3 | 5b-H4 | 6-H4 |
| Dongnong36 | 000 | 1a-H6 | 1b-H5 | 2a-H11 | / | 3a-H1 | 3b-H2 | 4-H2 | 5a-H3 | 5b-H2 | 6-H4 |
| Dongnong41 | 000 | 1a-H6 | 1b-H3 | 2a-H4 | 2b-H5 | 3a-H1 | 3b-H7 | 4-H2 | 5a-H3 | 5b-H4 | 6-H4 |
| Beidou 16 | 00 | 1a-H1 | 1b-H5 | 2a-H6 | 2b-H3 | 3a-H1 | 3b-H4 | 4-H2 | 5a-H2 | 5b-H1 | 6-H4 |
| Mengdou 11 | 00 | 1a-H6 | 1b-H3 | 2a-H6 | 2b-H3 | 3a-H1 | 3b-H4 | 4-H2 | 5a-H2 | 5b-H1 | 6-H4 |
| Dongnong 44 | 00 | 1a-H10 | / | / | 2b-H3 | 3a-H1 | 3b-H4 | 4-H1 | 5a-H2 | 5b-H1 | 6-H2 |
| McCall | 00 | 1a-H1 | 1b-H5 | 2a-H6 | 2b-H3 | 3a-H1 | 3b-H6 | 4-H2 | 5a-H1 | 5b-H5 | 6-H4 |
| Mengdou 32 | 00 | 1a-H6 | 1b-H3 | 2a-H6 | 2b-H3 | 3a-H1 | 3b-H4 | 4-H2 | 5a-H2 | 5b-H1 | 6-H4 |
| Maple Ridge | 00 | 1a-H1 | 1b-H5 | 2a-H6 | 2b-H3 | 3a-H1 | 3b-H2 | 4-H2 | 5a-H1 | 5b-H1 | 6-H4 |
| Daksoy | 00 | 1a-H1 | / | 2a-H6 | 2b-H3 | 3a-H1 | 3b-H4 | 4-H2 | 5a-H3 | 5b-H1 | 6-H4 |
| Agassiz | 00 | 1a-H6 | 1b-H3 | 2a-H6 | 2b-H3 | 3a-H1 | 3b-H6 | 4-H2 | 5a-H1 | 5b-H5 | 6-H4 |
| Canatto | 00 | 1a-H2 | 1b-H6 | 2a-H6 | 2b-H3 | 3a-H1 | 3b-H4 | 4-H4 | 5a-H2 | 5b-H1 | 6-H6 |
| Heihe 18 | 0 | 1a-H1 | 1b-H5 | 2a-H6 | 2b-H3 | 3a-H2 | 3b-H5 | 4-H1 | 5a-H4 | 5b-H2 | 6-H5 |
| Hefeng 25 | 0 | 1a-H10 | / | 2a-H6 | 2b-H3 | 3a-H1 | 3b-H4 | 4-H2 | 5a-H2 | 5b-H1 | 6-H4 |
| Heihe 43 | 0 | 1a-H1 | 1b-H5 | 2a-H6 | 2b-H3 | 3a-H2 | 3b-H5 | 4-H1 | 5a-H4 | 5b-H3 | 6-H2 |
| Heihe 27 | 0 | 1a-H10 | / | 2a-H6 | 2b-H3 | 3a-H1 | 3b-H4 | 4-H1 | 5a-H2 | 5b-H1 | 6-H2 |
| Dengke 1 | 0 | 1a-H1 | 1b-H5 | 2a-H6 | 2b-H3 | 3a-H1 | 3b-H4 | 4-H2 | 5a-H2 | 5b-H1 | 6-H4 |
| Beidou 37 | 0 | 1a-H10 | / | 2a-H6 | 2b-H3 | 3a-H1 | 3b-H4 | 4-H2 | 5a-H2 | 5b-H1 | 6-H4 |
| Fengshou 12 | 0 | 1a-H1 | 1b-H5 | 2a-H6 | 2b-H3 | 3a-H1 | 3b-H4 | 4-H2 | 5a-H2 | 5b-H1 | 6-H4 |
| Traill | 0 | 1a-H1 | 1b-H3 | 2a-H6 | 2b-H4 | 3a-H1 | 3b-H7 | 4-H2 | 5a-H2 | 5b-H1 | 6-H4 |
| Dongnong 4 | 0 | 1a-H1 | 1b-H5 | 2a-H6 | 2b-H3 | 3a-H1 | 3b-H8 | 4-H2 | 5a-H2 | 5b-H1 | 6-H4 |
| Norpro | 0 | 1a-H8 | 1b-H3 | 2a-H6 | 2b-H3 | 3a-H1 | 3b-H4 | 4-H2 | 5a-H1 | 5b-H1 | 6-H4 |
| Jiangmodou 1 | 0 | 1a-H1 | 1b-H5 | 2a-H6 | 2b-H3 | 3a-H1 | 3b-H4 | 4-H2 | 5a-H2 | 5b-H1 | 6-H4 |
| Barnes | 0 | 1a-H1 | 1b-H6 | 2a-H6 | 2b-H3 | 3a-H1 | 3b-H4 | 4-H2 | 5a-H2 | 5b-H1 | 6-H4 |
| Dawson | 0 | 1a-H6 | 1b-H3 | 2a-H6 | 2b-H3 | 3a-H1 | 3b-H7 | 4-H2 | 5a-H2 | 5b-H5 | 6-H4 |
| Chico | 0 | 1a-H6 | 1b-H3 | 2a-H1 | 2b-H6 | 3a-H1 | 3b-H7 | 4-H2 | 5a-H2 | 5b-H5 | 6-H4 |
| Heinong 16 | I | 1a-H1 | 1b-H5 | 2a-H6 | 2b-H3 | 3a-H1 | 3b-H7 | 4-H2 | 5a-H2 | 5b-H5 | 6-H4 |
| Heinong 26 | I | / | 1b-H5 | / | 2b-H3 | 3a-H1 | 3b-H7 | 4-H2 | 5a-H3 | 5b-H4 | 6-H4 |
| Suinong 14 | I | 1a-H10 | / | 2a-H6 | 2b-H3 | 3a-H1 | 3b-H4 | 4-H1 | 5a-H2 | 5b-H1 | 6-H2 |
| Kato | I | 1a-H6 | 1b-H3 | 2a-H6 | 2b-H3 | 3a-H1 | 3b-H7 | 4-H2 | 5a-H2 | 5b-H1 | 6-H4 |
| Taixingheidou | I | 1a-H11 | / | 2a-H1 | 2b-H7 | 3a-H1 | 3b-H7 | 4-H3 | 5a-H3 | 5b-H4 | 6-H1 |
| Haroson | I | 1a-H6 | 1b-H3 | 2a-H6 | 2b-H3 | 3a-H1 | 3b-H4 | 4-H2 | 5a-H2 | 5b-H1 | 6-H4 |
| Tiefeng 19 | I | 1a-H3 | 1b-H1 | 2a-H6 | 2b-H3 | 3a-H1 | 3b-H7 | 4-H2 | 5a-H2 | / | 6-H4 |
| Parker | I | 1a-H6 | 1b-H3 | / | 2b-H3 | 3a-H1 | 3b-H3 | 4-H2 | 5a-H2 | 5b-H1 | 6-H4 |
| Granite | I | 1a-H2 | 1b-H6 | 2a-H6 | 2b-H4 | 3a-H1 | 3b-H4 | 4-H2 | 5a-H2 | 5b-H1 | 6-H4 |
| NE1900 | I | 1a-H3 | 1b-H1 | 2a-H6 | 2b-H3 | 3a-H1 | 3b-H4 | 4-H2 | 5a-H3 | 5b-H1 | 6-H4 |
| Jilin 20 | II | 1a-H1 | 1b-H5 | 2a-H6 | 2b-H4 | 3a-H1 | 3b-H2 | 4-H3 | 5a-H3 | 5b-H2 | 6-H1 |
| Holt | II | 1a-H3 | 1b-H1 | 2a-H6 | 2b-H3 | 3a-H1 | 3b-H4 | 4-H2 | 5a-H1 | 5b-H1 | 6-H4 |
| Century 84 | II | 1a-H1 | 1b-H5 | 2a-H6 | 2b-H3 | 3a-H1 | 3b-H1 | 4-H2 | 5a-H3 | 5b-H2 | 6-H4 |
| Olympus | II | 1a-H3 | 1b-H1 | 2a-H6 | 2b-H4 | 3a-H1 | 3b-H4 | 4-H2 | 5a-H3 | 5b-H1 | 6-H4 |
| LN92-7369 | II | 1a-H1 | 1b-H5 | 2a-H6 | 2b-H3 | 3a-H1 | 3b-H4 | 4-H2 | 5a-H3 | 5b-H1 | 6-H4 |
| IL1 | II | 1a-H7 | 1b-H3 | 2a-H4 | 2b-H5 | 3a-H3 | 3b-H4 | 4-H3 | 5a-H5 | 5b-H1 | 6-H1 |
| Yongchengzihuadou | II | 1a-H11 | / | 2a-H1 | 2b-H7 | 3a-H1 | 3b-H2 | 4-H2 | 5a-H3 | 5b-H2 | 6-H4 |
| Xiangchundou 24 | II | 1a-H10 | / | 2a-H10 | 2b-H1 | 3a-H1 | 3b-H7 | 4-H3 | 5a-H3 | / | 6-H1 |
| Tiefeng 33 | III | 1a-H1 | 1b-H5 | 2a-H6 | 2b-H3 | 3a-H1 | 3b-H2 | 4-H2 | 5a-H2 | / | 6-H4 |
| Tiefeng 31 | III | 1a-H1 | 1b-H5 | 2a-H6 | 2b-H4 | 3a-H1 | 3b-H2 | 4-H2 | 5a-H1 | 5b-H2 | 6-H4 |
| Jindou 19 | III | / | 1b-H6 | 2a-H6 | / | / | / | 4-H2 | 5a-H3 | / | 6-H3 |
| Athow | III | 1a-H1 | 1b-H6 | 2a-H6 | 2b-H4 | 3a-H1 | 3b-H4 | 4-H2 | 5a-H2 | 5b-H1 | 6-H4 |
| Zhonghuang30 | III | 1a-H1 | 1b-H5 | 2a-H6 | 2b-H4 | 3a-H1 | 3b-H4 | 4-H2 | 5a-H1 | 5b-H1 | 6-H4 |
| KS3494 | III | 1a-H1 | 1b-H5 | 2a-H6 | 2b-H4 | 3a-H1 | 3b-H7 | 4-H2 | 5a-H1 | 5b-H5 | 6-H4 |
| LN89-5699 | III | 1a-H1 | 1b-H5 | 2a-H6 | 2b-H4 | 3a-H1 | 3b-H4 | 4-H2 | 5a-H1 | 5b-H1 | 6-H4 |
| Williams 82 | III | 1a-H1 | 1b-H5 | 2a-H6 | 2b-H4 | 3a-H1 | 3b-H4 | 4-H2 | 5a-H1 | 5b-H1 | 6-H4 |
| Xudou 9 | III | 1a-H1 | 1b-H5 | 2a-H6 | 2b-H3 | 3a-H1 | 3b-H4 | 4-H2 | 5a-H2 | 5b-H1 | 6-H4 |
| Zhongdou 39 | III | 1a-H10 | / | 2a-H10 | 2b-H1 | 3a-H1 | 3b-H7 | 4-H2 | 5a-H2 | 5b-H4 | 6-H4 |
| Zhonghuang13 | III | 1a-H1 | 1b-H5 | 2a-H4 | 2b-H6 | 3a-H1 | 3b-H7 | 4-H3 | 5a-H3 | 5b-H4 | 6-H1 |
| Huaidou 9 | III | 1a-H11 | hap7 | 2a-H6 | 2b-H4 | 3a-H1 | 3b-H7 | 4-H3 | 5a-H3 | 5b-H4 | 6-H1 |
| IL2 | III | 1a-H3 | 1b-H1 | 2a-H3 | 2b-H6 | 3a-H1 | 3b-H4 | 4-H2 | 5a-H1 | 5b-H1 | 6-H4 |
| Huachun 6 | III | 1a-H10 | / | 2a-H1 | 2b-H7 | 3a-H1 | 3b-H2 | 4-H2 | 5a-H3 | 5b-H2 | 6-H4 |
| Flyer | IV | 1a-H1 | 1b-H5 | 2a-H6 | 2b-H4 | 3a-H1 | 3b-H7 | 4-H2 | 5a-H1 | 5b-H5 | 6-H4 |
| Omaha | IV | 1a-H1 | 1b-H5 | 2a-H3 | 2b-H7 | 3a-H1 | 3b-H3 | 4-H2 | 5a-H1 | 5b-H1 | 6-H4 |
| CF461 | IV | 1a-H1 | 1b-H5 | 2a-H6 | 2b-H7 | 3a-H1 | 3b-H4 | 4-H2 | 5a-H1 | 5b-H1 | 6-H4 |
| Zheng 92116 | IV | 1a-H3 | 1b-H1 | 2a-H6 | 2b-H4 | 3a-H1 | 3b-H2 | 4-H2 | 5a-H3 | 5b-H2 | 6-H4 |
| Jindou 39 | IV | 1a-H3 | 1b-H2 | 2a-H6 | 2b-H3 | 3a-H1 | 3b-H2 | 4-H3 | 5a-H3 | / | 6-H1 |
| Shanning 16 | IV | 1a-H1 | 1b-H5 | 2a-H6 | 2b-H4 | 3a-H1 | / | 4-H3 | 5a-H3 | / | / |
| Calhoun | IV | 1a-H6 | 1b-H3 | 2a-H1 | 2b-H6 | 3a-H1 | 3b-H6 | 4-H2 | 5a-H3 | 5b-H4 | 6-H4 |
| Guandou 2 | IV | 1a-H3 | 1b-H1 | 2a-H6 | 2b-H4 | 3a-H1 | 3b-H2 | 4-H3 | 5a-H3 | 5b-H2 | 6-H1 |
| UA 4805 | IV | 1a-H4 | 1b-H1 | 2a-H6 | 2b-H4 | 3a-H1 | 3b-H4 | 4-H2 | 5a-H3 | 5b-H1 | 6-H4 |
| Houzimao | IV | 1a-H1 | 1b-H5 | 2a-H1 | 2b-H7 | 3a-H1 | / | 4-H1 | 5a-H3 | 5b-H5 | 6-H2 |
| Nathan | V | 1a-H1 | 1b-H5 | 2a-H6 | 2b-H4 | 3a-H1 | 3b-H4 | 4-H2 | 5a-H3 | 5b-H1 | 6-H4 |
| Holladay | V | 1a-H11 | 1b-H8 | 2a-H6 | 2b-H4 | 3a-H1 | 3b-H7 | 4-H2 | 5a-H3 | 5b-H5 | 6-H4 |
| Hutcheson | V | / | 1b-H1 | 2a-H6 | 2b-H4 | 3a-H1 | 3b-H6 | 4-H2 | 5a-H3 | 5b-H5 | 6-H4 |
| R01-3474F | V | 1a-H3 | 1b-H1 | 2a-H6 | 2b-H4 | 3a-H1 | 3b-H2 | 4-H2 | 5a-H3 | 5b-H1 | 6-H4 |
| Dian 86-4 | V | / | 1b-H5 | 2a-H1 | 2b-H7 | 3a-H1 | 3b-H7 | 4-H1 | 5a-H3 | / | 6-H2 |
| TN04-5321 | V | 1a-H1 | / | 2a-H6 | 2b-H4 | 3a-H1 | 3b-H2 | 4-H2 | 5a-H3 | / | 6-H4 |
| Shangdou 14 | V | 1a-H1 | 1b-H5 | 2a-H7 | 2b-H9 | 3a-H1 | 3b-H2 | 4-H2 | 5a-H3 | 5b-H2 | 6-H4 |
| Diandou 7 | V | 1a-H1 | 1b-H5 | 2a-H6 | 2b-H4 | 3a-H1 | / | 4-H2 | 5a-H1 | 5b-H2 | 6-H4 |
| Desha | VI | 1a-H3 | 1b-H1 | 2a-H6 | 2b-H4 | 3a-H1 | 3b-H4 | 4-H2 | 5a-H3 | 5b-H1 | 6-H4 |
| Boggs | VI | 1a-H2 | 1b-H5 | 2a-H6 | 2b-H4 | 3a-H1 | 3b-H2 | 4-H2 | 5a-H3 | 5b-H2 | 6-H4 |
| Suxiandou 19 | VI | 1a-H3 | / | 2a-H5 | / | 3a-H1 | / | 4-H3 | 5a-H3 | / | 6-H1 |
| G01-PR16 | VI | 1a-H11 | / | / | / | 3a-H1 | 3b-H6 | 4-H2 | 5a-H3 | 5b-H5 | 6-H4 |
| Zhongdou 38 | VI | / | 1b-H1 | 2a-H6 | 2b-H4 | 3a-H1 | 3b-H2 | 4-H2 | 5a-H3 | / | 6-H4 |
| Musen | VI | 1a-H9 | hap7 | 2a-H6 | 2b-H4 | 3a-H1 | 3b-H9 | 4-H2 | 5a-H3 | 5b-H5 | 6-H4 |
| D95-6271 | VI | 1a-H3 | 1b-H1 | 2a-H3 | 2b-H6 | 3a-H1 | 3b-H1 | 4-H3 | 5a-H3 | 5b-H2 | 6-H1 |
| Wuhuasiyuehuang | VI | 1a-H3 | / | / | 2b-H7 | 3a-H1 | / | 4-H2 | 5a-H3 | 5b-H2 | 6-H4 |
| Nannong 493/1 | VI | 1a-H11 | / | 2a-H2 | 2b-H7 | 3a-H1 | 3b-H2 | 4-H3 | 5a-H3 | 5b-H2 | 6-H1 |
| Hengyangbayueqing | VI | 1a-H11 | 1b-H5 | / | 2b-H1 | 3a-H2 | 3b-H7 | 4-H3 | 5a-H3 | 5b-H4 | 6-H1 |
| Benning | VII | 1a-H3 | 1b-H1 | 2a-H6 | 2b-H4 | 3a-H1 | 3b-H2 | 4-H2 | 5a-H3 | 5b-H2 | 6-H4 |
| Santee | VII | 1a-H12 | hap7 | 2a-H6 | 2b-H4 | 3a-H1 | 3b-H2 | 4-H2 | 5a-H3 | 5b-H2 | 6-H4 |
| Stonewall | VII | 1a-H3 | 1b-H1 | 2a-H6 | 2b-H4 | / | 3b-H2 | 4-H2 | 5a-H3 | 5b-H2 | 6-H4 |
| Hagood | VII | 1a-H11 | / | 2a-H3 | 2b-H6 | 3a-H1 | 3b-H7 | 4-H2 | 5a-H3 | 5b-H5 | 6-H4 |
| Nanxiadou 25 | VII | 1a-H1 | 1b-H5 | 2a-H1 | 2b-H7 | 3a-H1 | / | 4-H2 | 5a-H3 | / | 6-H4 |
| Tongshanbaopihuang | VII | 1a-H6 | 1b-H4 | 2a-H1 | 2b-H7 | 3a-H1 | / | 4-H3 | 5a-H3 | / | 6-H1 |
| Huangfengwo | VII | 1a-H3 | 1b-H1 | 2a-H1 | 2b-H7 | 3a-H1 | 3b-H7 | 4-H1 | 5a-H3 | 5b-H5 | 6-H2 |
| Dowling | VIII | 1a-H9 | hap7 | 2a-H6 | 2b-H4 | 3a-H1 | 3b-H2 | 4-H2 | 5a-H3 | 5b-H2 | 6-H4 |
| Motte | VIII | 1a-H1 | hap7 | 2a-H6 | 2b-H4 | 3a-H1 | 3b-H6 | 4-H2 | 5a-H3 | 5b-H5 | 6-H4 |
| Prichard | VIII | 1a-H1 | 1b-H5 | 2a-H6 | 2b-H4 | 3a-H1 | / | 4-H2 | 5a-H3 | 5b-H2 | 6-H4 |
| Aijiaoqing | VIII | 1a-H10 | / | 2a-H1 | / | 3a-H1 | / | 4-H3 | 5a-H3 | 5b-H5 | 6-H1 |
| Nandou 12 | VIII | 1a-H6 | 1b-H3 | 2a-H1 | 2b-H7 | 3a-H1 | / | 4-H3 | 5a-H3 | 5b-H5 | 6-H1 |
| Lanxidaqingdou | VIII | 1a-H1 | 1b-H5 | 2a-H10 | 2b-H1 | 3a-H1 | / | 4-H2 | 5a-H3 | / | 6-H4 |
| Shangraodaqingsi | VIII | 1a-H1 | 1b-H5 | 2a-H12 | 2b-H1 | 3a-H1 | / | 4-H3 | 5a-H3 | 5b-H2 | 6-H1 |
| Nandou 17 | VIII | 1a-H1 | 1b-H5 | 2a-H1 | 2b-H7 | 3a-H1 | / | 4-H2 | 5a-H3 | 5b-H4 | 6-H4 |
| Guixia 3 | VIII | 1a-H10 | / | 2a-H8 | 2b-H2 | 3a-H1 | 3b-H7 | 4-H3 | 5a-H3 | 5b-H4 | 6-H1 |
| Pinguohuangdou | VIII | 1a-H1 | 1b-H5 | 2a-H1 | 2b-H7 | 3a-H1 | / | 4-H3 | 5a-H3 | / | 6-H1 |
| Qiudou 1 | VIII | 1a-H10 | / | 2a-H1 | 2b-H7 | 3a-H1 | / | 4-H2 | 5a-H3 | 5b-H4 | 6-H4 |
| Zigongdongdou | VIII | / | / | 2a-H10 | 2b-H1 | 3a-H1 | 3b-H2 | 4-H3 | 5a-H3 | / | 6-H1 |
| IAC-8 | IX | 1a-H1 | 1b-H5 | 2a-H9 | 2b-H4 | 3a-H1 | 3b-H2 | 4-H2 | 5a-H3 | 5b-H2 | 6-H4 |
| UFV-3 | IX | 1a-H3 | 1b-H1 | 2a-H10 | 2b-H1 | 3a-H1 | 3b-H2 | 4-H2 | 5a-H3 | 5b-H2 | 6-H4 |
| FT-15 | IX | 1a-H3 | / | 2a-H5 | 2b-H6 | / | / | 4-H2 | 5a-H3 | / | 6-H4 |
| Alamo | IX | 1a-H1 | 1b-H5 | 2a-H9 | 2b-H1 | 3a-H1 | 3b-H2 | 4-H3 | 5a-H3 | / | / |
| I.C. 192 | IX | 1a-H11 | 1b-H3 | 2a-H10 | 2b-H1 | 3a-H1 | 3b-H6 | 4-H2 | 5a-H3 | 5b-H4 | 6-H4 |
| Jupiter | IX | 1a-H1 | 1b-H5 | 2a-H9 | / | 3a-H1 | 3b-H2 | 4-H2 | 5a-H3 | / | 6-H4 |
| CIGRAS-06 | X | 1a-H11 | hap7 | 2a-H6 | 2b-H4 | 3a-H1 | 3b-H1 | 4-H2 | 5a-H3 | 5b-H2 | 6-H4 |
| CIGRAS-51 | X | 1a-H11 | hap7 | 2a-H9 | 2b-H1 | 3a-H1 | 3b-H2 | 4-H2 | 5a-H3 | 5b-H2 | 6-H4 |
| Jiangledaqingdou | X | 1a-H10 | / | 2a-H1 | 2b-H7 | 3a-H1 | / | 4-H3 | 5a-H3 | 5b-H4 | 6-H1 |

Note: “/” represents that the Variety was not sequenced.
