# Supplementary material for: Natural variations of FT family genes in soybean varieties covering a wide range of maturity groups
Source: BMC Genomics. 2019 Mar 20;20:230. doi: 10.1186/s12864-019-5577-5 (PMC6425728; doi:10.1186/s12864-019-5577-5)
Supplement: Supplementary file 8 — Table S8. Polymorphic sites used for defining the haplotypes of the 10 soybean FT family genes. (DOCX 14 kb) [file 12864_2019_5577_MOESM8_ESM.docx]

**Table S8. Polymorphic sites used for defining the haplotypes of the 10 soybean *FT* family genes**

| **Gene** | **Polymorphic sites used for defining haplotypes** |
| --- | --- |
| *GmFT1a* | s5227,s5298,s5322,s5323,s5324,s5344,s5373 |
| *GmFT1b* | s125,s126, s220,s387,s421,s627,s644,s670, Indel730, s736,,s775, **Indel790**, s805, Indel857, Indel869,s943, s990,s1083,s1108,s1162,s1198,s1243,s1301,Indel1318, s1326,s1351 , Indel1364,s1373,s1378,s1379,s1415,**s1423**,s1433,s1473,s1536,s1551,s1553,s1582,**Indel1593**,  Indel1636,**Indel1674**, s1683,**s1684**, s1707,s1731,s1787,s1867,s1888,s1889,**Indel1914**, s2075,  s2077,s2094,s2111,Indel2130,s2139,s2161,s2174,s2178,**s2200**,s2248,s2264,s2311,s2364,**s2380**, **s2388**,s2396,**Indel2409**,**s2443**,s2491,**s2492**,**s2570**,**s2618**,**s2634**,s2673,s2674 |
| *GmFT2a* | **s125**,**s350**,**s371**,**s454**,s459,**s766**,Indel997,s1039,**s1048**,s1355,s1521,**Indel1539**,s1621,s1794,  Indel1848,s2069,**Indel2874**,s3253,**s3261**,s3675,Indel3787,s3833,**Indel3841**,s4074,**s4082**,**s4269**,  s4276,**s4307**,s4335,**s4420**,**s4645**,**s4671**,s4943,s5365 |
| *GmFT2b* | **s189**,**s222**,**s755**,s762,s867,Indel883,**Indel893**,**Indel992**,s1099,s1148,s1180,s1232,**s1343**,s1384,Indel1386,**s1456**,s1629,s1654, Indel1692, Indel1731,s1948,**Indel1956**,s1961,s2073,Indel2143,s2213, s2319, s2579,s2587,Indel2588,s2612,s2633, s2637, s2702,**s2711**,s2826,**s2904**,**s2908**, **s3040** |
| *GmFT3a* | Indel712,s822,**s1794**,s1953,s2129,s2259,**s2301** |
| *GmFT3b* | Indel521,**s547**,s581,s588,s596,Indel839,**s866**,s876,s908,s1167,**s1349**,s1453,**s1764**,**s1774**,**s1874**,**s1963,s2033** |
| *GmFT4* | **Indel560**,**s580**,**Indel1278**,**Indel1351** |
| *GmFT5a* | **Indel68**,**S1129**,S1187,**S1487**,**Indel1577**,**Indel1914** |
| *GmFT5b* | s62,s80,**s160**,Indel346,s539,s553,s554,**s569**,Indel587,s707,s1032,**s1117**,s1163,s1344,s1558, s1662,  **s1821**,s1856,**Indel1877**,s2194,s2418,s2435 |
| *GmFT6* | **s74**,**s754**,s1144,s1450,**s4924**,**s5016** |

Note: Bold font represents critical polymorphic sites used for defining tagging haplotype.
