# Supplementary material for: Natural variations of FT family genes in soybean varieties covering a wide range of maturity groups
Source: BMC Genomics. 2019 Mar 20;20:230. doi: 10.1186/s12864-019-5577-5 (PMC6425728; doi:10.1186/s12864-019-5577-5)
Supplement: Supplementary file 9 — Table S9. Polymorphic site features used for defining the haplotypes of the 10 soybean FT family genes. (DOCX 16 kb) [file 12864_2019_5577_MOESM9_ESM.docx]

**Table S9. Polymorphic site features used for defining the haplotypes of the 10 soybean *FT* family genes**

| **Gene** | **No.of sites used for haplotype** | **Types of sites** | **No.of sites used for tagging haplotype** |
| --- | --- | --- | --- |
| *GmFT1a* | 7 | 7 SNPs | / |
| *GmFT1b* | 76 | 64 SNPs,12 Indels | 15 |
| *GmFT2a* | 34 | 28 SNPs,6 Indels | 16 |
| *GmFT2b* | 39 | 30 SNPs,9 Indels | 13 |
| *GmFT3a* | 7 | 6 SNPs, 1 Indel | 2 |
| *GmFT3b* | 17 | 15 SNPs,2 Indels | 8 |
| *GmFT4* | 4 | 1 SNP, 3 Indels | 4 |
| *GmFT5a* | 6 | 3 SNPs, 3 Indels | 5 |
| *GmFT5b* | 23 | 20 SNPs, 3 Indels | 5 |
| *GmFT6* | 6 | 6 SNPs | 4 |
