# Supplementary material for: Natural variations of FT family genes in soybean varieties covering a wide range of maturity groups
Source: BMC Genomics. 2019 Mar 20;20:230. doi: 10.1186/s12864-019-5577-5 (PMC6425728; doi:10.1186/s12864-019-5577-5)
Supplement: Supplementary file 10 — Table S10. Different types of soybean FT proteins and their haplotype distribution. (DOCX 18 kb) [file 12864_2019_5577_MOESM10_ESM.docx]

**Table S10. Different types of soybean FT proteins and their haplotype distribution**

| **Gene** | **Types of Protein** | | **Amino acid in mutational site** | **Haplotype distribution** | |
| --- | --- | --- | --- | --- | --- |
| *GmFT1a* | P0 | I_105_R_129_G_137_W_138_G_154_ | | 1a-H1,1a-H9,1a-H11 |  |
|  | P1 | I_105_R_129_G_137_L_138_G_154_ | | 1a-H2 |  |
|  | P2 | I_105_P_129_G_137_W_138_E_154_ | | 1a-H3 |  |
|  | P3 | I_105_P_129_G_137_R_138_E_154_ | | 1a-H4 |  |
|  | P4 | M_105_R_129_G_137_W_138_G_154_ | | 1a-H5,1a-H6,1a-H8 |  |
|  | P5 | I_105_R_129_A_137_W_138_G_154_ | | 1a-H10 |  |
|  | P6 | I_105_P_129_G_137_W_138_G_154_ | | 1a-H12 |  |
| *GmFT1b* | P0 | R_122_N_148_N_164_D_169_ | | 1b-H5,1b-H6 |  |
|  | P1 | R_122_N_148_N_164_A_169_ | | 1b-H1,1b-H2 |  |
|  | P2 | R_122_D_148_H_164_D_169_ | | 1b-H3,1b-H4 |  |
|  | P3 | S_122_N_148_N_164_D_169_ | | 1b-H7 |  |
|  | P4 | S_122_N_148_N_164_A_169_ | | 1b-H8 |  |
| *GmFT2a* | P0 | E_23_G_169_ | | 2a-H1,2a-H2,2a-H4,2a-H5,2a-H6,2a-H7, 2a-H8,2a-H10,2a-H11,2a-H12 |  |
|  | P1 | D_23_G_169_ | | 2a-H3 |  |
|  | P2 | E_23_D_169_ | | 2a-H9 |  |
| *GmFT2b* | P0 | R_126_ | | 2b-H1,2b-H2,2b-H3,2b-H4,2b-H5,2b-H8, 2b-H9 |  |
|  | P1 | H_126_ | | 2b-H6,2b-H7 |  |
| *GmFT3b* | P0 | S_107_ | | 3b-H2,3b-H3,3b-H4,3b-H5,3b-H6,3b-H7, 3b-H8,3b-H9 |  |
|  | P1 | N_107_ | | 3b-H1 |  |
| *GmFT5b* | P0 | T_27_ | | 5b-H1 |  |
|  | P1 | A_27_ | | 5b-H2,5b-H3,5b-H4,5b-H5 |  |

Note: I_105_,subscript 105 represents the position of this amino acid of soybean FT protein sequence, others are the same as I_105_.
